# Targeting C/EBPα overcomes primary resistance and improves the efficacy of FLT3 inhibitors in acute myeloid leukaemia

**DOI:** 10.1038/s41467-023-37381-4

**Published:** 2023-04-05

**Authors:** Hanlin Wang, Guanghao Luo, Xiaobei Hu, Gaoya Xu, Tao Wang, Minmin Liu, Xiaohui Qiu, Jianan Li, Jingfeng Fu, Bo Feng, Yutong Tu, Weijuan Kan, Chang Wang, Ran Xu, Yubo Zhou, Jianmin Yang, Jia Li

**Affiliations:** 1 State key Laboratory of Drug Research, Shanghai Institute of Materia Medica, Chinese Academy of Sciences, Shanghai, 201203 China; 2 College of Pharmacy, Fudan University, Shanghai, 210023 China; 3 University of Chinese Academy of Sciences, Beijing, 100049 China; 4 School of Pharmaceutical Science and Technology, Hangzhou Institute for Advanced Study, University of Chinese Academy of Sciences, Hangzhou, 310000 China; 5 Zhongshan Institute for Drug Discovery, Shanghai Institute of Materia Medica, Chinese Academy of Sciences, Guangdong, 528400 China; 6 School of Chinese Materia Medica, Nanjing University of Chinese Medicine, Nanjing, 210023 China; 7 Department of Hematology, Changhai Hospital, Naval Medical University, Shanghai, 200433 China; 8 School of Pharmaceutical Science, Jiangnan University, Wuxi, 214122 China; 9 School of Life Science and Biopharmaceutics, Shenyang Pharmaceutical University, No.103 Wenhua Road, Shenyang, Liaoning China

**Keywords:** Myeloma, Cancer therapeutic resistance, Tumour biomarkers, Cancer genomics

## Abstract

The outcomes of *FLT3-ITD* acute myeloid leukaemia (AML) have been improved since the approval of FLT3 inhibitors (FLT3i). However, approximately 30-50% of patients exhibit primary resistance (PR) to FLT3i with poorly defined mechanisms, posing a pressing clinical unmet need. Here, we identify C/EBPα activation as a top PR feature by analyzing data from primary AML patient samples in Vizome. C/EBPα activation limit FLT3i efficacy, while its inactivation synergistically enhances FLT3i action in cellular and female animal models. We then perform an in silico screen and identify that guanfacine, an antihypertensive medication, mimics C/EBPα inactivation. Furthermore, guanfacine exerts a synergistic effect with FLT3i in vitro and in vivo. Finally, we ascertain the role of C/EBPα activation in PR in an independent cohort of *FLT3-ITD* patients. These findings highlight C/EBPα activation as a targetable PR mechanism and support clinical studies aimed at testing the combination of guanfacine with FLT3i in overcoming PR and enhancing the efficacy of FLT3i therapy.

## Abstract (web summary)

Resistance of FLT3-ITD acute myeloid leukaemia (AML) patients to FLT3 inhibitors (FLT3i) remains an urgent clinical challenge. Here, the authors identify C/EBPα activation as a mechanism of FLT3i resistance and therapeutically target C/EBPα activation in combination with FLT3i in preclinical models FLT3-ITD AML.

## Introduction

*FLT3-ITD* is the most common driver mutation, with approximately 30% in acute myeloid leukaemia (AML), and is associated with poor clinical outcomes^1–3^. Mechanistically, ITD mutation results in constitutive activation of FLT3 signalling, which activates downstream kinases, including MAPK/ERK, JAK/STAT, and AKT, enhances cellular proliferation, and blocks apoptosis and differentiation in AML with *FLT3* mutations^4^.

FLT3 inhibitors (FLT3i), including midostaurin (first generation), gilteritinib and quizartinib (second generation), have achieved great success in the treatment of *FLT3*-mutated AML^5–10^. Based on their binding to different conformations of the FLT3 receptor, FLT3i can be classified into 2 types. Type I inhibitors (midostaurin, gilteritinib and crenolanib) bind to the active receptor conformation and inhibit FLT3 signalling in AML cells with ITD and/or TKD mutations. Type II inhibitors (quizartinib, sorafenib and ponatinib) bind to the inactive conformation of the FLT3 receptor, showing significant clinical activity in *FLT3-ITD* but not in *FLT3-TKD* AML^9^. Importantly, approximately 30-50% of *FLT3-ITD* patients show primary resistance (PR) to type I and type II inhibitors^8,10–12^, making PR a pressing unmet clinical need^13,14^. However, the PR mechanisms of FLT3i are poorly defined, and it remains a major challenge to identify therapeutic strategies to overcome PR and enhance FLT3i efficacy^15^.

Recent studies in AML cell lines have reported that knocking out ATM^16^, SPRY3^17^, or regulators of the MAPK and mTOR pathways^18^ confers FLT3i resistance. However, the genes and pathways are not validated using primary AML patient samples. Here, we perform both bioinformatics and experimental analyzes on *FLT3-ITD* AML patient samples to determine the phenotypic, genomic and transcriptional PR features. Our work reveal C/EBPα activation as the top PR feature for both type I and type II FLT3i. Genetic or pharmacological downregulation of C/EBPα activity results in enhanced sensitivity of AML cells to FLT3i, highlighting C/EBPα activation as a targetable PR mechanism and supporting clinical studies evaluating combination therapy of FLT3i and C/EBPα inactivation as a next-generation AML treatment strategy.

## Results

### C/EBPα as the top affected target in FLT3i-resistant patients

**Fig. 1:**
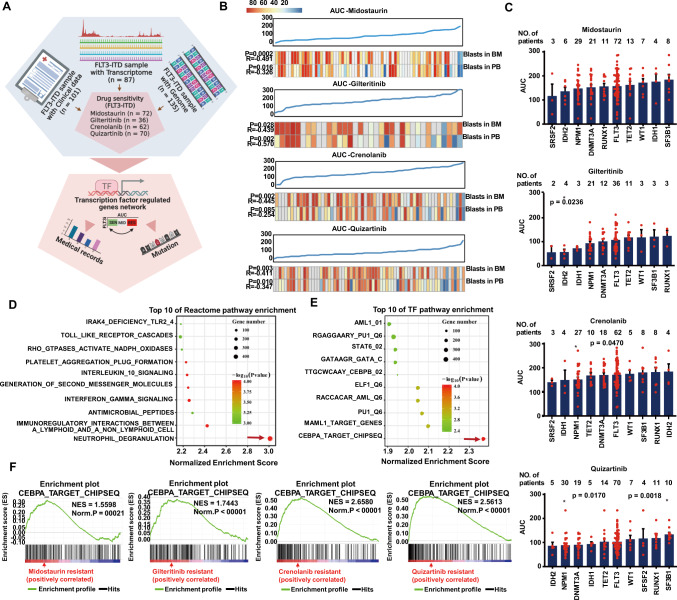
Identification of potential key factors for FLT3i resistance. **A** Simplified schematic integrating patient clinical, WES, RNA-seq and FLT3i in vitro screening data to identify biomarkers predicting FLT3i response. **B** AUCs of four FLT3i from primary AML samples in Vizome (gilteritinib, *n* = 30; quizartinib, *n* = 56; midostaurin, *n* = 60; crenolanib, *n* = 54) were compared with blasts (%) in bone marrow (BM) and peripheral blood (PB). Significance was determined by the Spearman method. *P* < 0.05 represented significance, and *R* < 0 represented a negative correlation. The Y-axis indicates the AUC of the patient sample to FLT3i. **C** The AUCs of four FLT3i in AML common somatic mutation groups. AUCs in per group are presented as mean ± SEM. Significance was determined by the two-sided Wilcoxon *rank sum* test. Common differentially expressed reactome signalling pathways (**D**) and transcription factors (TFs) determined by GSEA (**E**). GSEA enrichment profiles (included p-value and enrichment score) of C/EBPα target genes in four FLT3i-resistant *FLT3-ITD* AML patients (**F**). Exact *p* values in Source Data file. Data for all graphs in Source Data file.

To identify targets involved in FLT3i resistance, we analyzed patient samples from the Beat AML cohort^19^, which had been subjected to small molecule inhibitor (midostaurin, gilteritinib, crenolanib or quizartinib) screening and had detailed clinical annotations, whole exome sequencing (WES), RNA-seq data and in vitro sensitivity data (Fig. 1A). We compared the area under the curve (AUC) distribution of drug sensitivity to the clinical characteristics and found a significant association between FLT3i resistance and blast counts in bone marrow and peripheral blood, indicating that AML samples with high blast counts were more sensitive to FLT3i (Fig. 1B). However, there was no significant correlation between WBC count and drug sensitivity (Supplementary Fig. 1).

We also evaluated the relationship between the AUC of drug sensitivity and common somatic mutations in AML (Fig. 1C) and observed a lower AUC of samples with *NPM1* mutation to the various FLT3 inhibitors, such as crenolanib (*P* < 0.01), quizartinib (*P* < 0.01), and gilteritinib (*P* = 0.07) (Fig. 1C). *NPM1* mutations accounted for 41% of *FLT3-ITD* patients (Supplementary Fig. 2). We divided samples of the bottom tertile AUC distribution as the sensitive (S) group and the top tertile as the resistant (R) group. We used the combined gene set enrichment analysis (GSEA) of the RNA-seq data to define a rank-based gene expression signature (signal2noise score) for each S and R group of 4 FLT3i and then determined the average rank for each gene to define an average-rank signature by GSEA (Supplementary Data 1). We also compared the differential expression of genes (DEGs) in the S and R groups of the four FLT3i groups and found a total of 528, 114, 183 and 324 DEGs with fold changes ≥ 2 or ≤ 0.5 and a false discovery rate (FDR) ≤ 0.05, respectively (Supplementary Data 2). Enriched signalling pathways from the above two methods were analyzed by GSEA and Metascape based on databases, including KEGG, Reactome, and GObp, where neutrophil degranulation (ND) and regulation of cytokine production pathways were most significantly enriched in all four differential gene sets (Fig. 1D and Supplementary Fig. 3A). GSEA identified C/EBPα as the top common activated transcription factor (TF) in all four FLT3i R groups based on TF target v7.4 databases (Fig. 1E, F, Supplementary Fig. 3B). Moreover, we found that the genes regulated by C/EBPα were mainly enriched in pathways related to leucocyte differentiation, ND and immune response by Metascape (Supplementary Fig. 3C). These findings indicate that C/EBPα is a potential target in FLT3i-resistant patients.

### C/EBPα activation confers FLT3i resistance

**Fig. 2:**
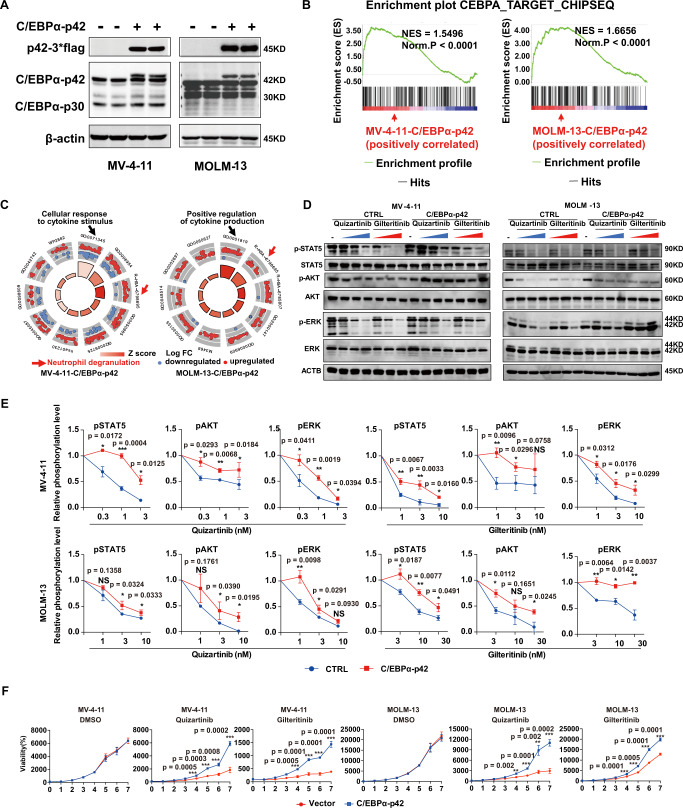
Overexpression of C/EBPα-p42 reduces the sensitivity of *FLT3 ITD* AML cells to FLT3i in vitro. **A** C/EBPα-p42 was overexpressed in MV-4-11 and MOLM-13 cell lines, as confirmed by western blotting. *n* = 3 independent experiments. **B** GSEA plots of the C/EBPα-regulated genes in C/EBPα-p42-overexpressing MV-4-11 and MOLM-13 cells. **C** Functional enrichment analysis of the differential signalling pathways in C/EBPα-p42-overexpressing MV-4-11 and MOLM-13 cells. **D** Changes in STAT5, AKT and ERK phosphorylation in MV-4-11 and MOLM-13 cells with or without C/EBPα-p42 overexpression under quizatinib (0.3, 1, or 3 nM) or gilteritinib (1, 3 or 10 nM) in MV-4-11 and quizatinib (1, 3, or 10 nM) or gilteritinib (3, 10, or 30 nM) in MOLM-13 for 2 h. **E** Quantification of the phosphorylation changes in (**D**). *n* = 3 independent experiments. Data are Means ± SEM. Significance was analyzed by equal variance two-tailed *t* test.*P < 0.05, **P < 0.01, ***P < 0.001. **F** Growth curve changes in MV-4-11 and MOLM-13 cells with or without C/EBPα-p42 overexpression treated with DMSO, quizartinib or gilteritinib. *n* = 3 independent experiments. Data are Means ± SEM. Significance was analyzed by equal variance two-tailed *t* test.*P < 0.05, **P < 0.01, ***P < 0.001. Exact *p* values in Source Data file. Data for all graphs in Source Data file.

**Fig. 3:**
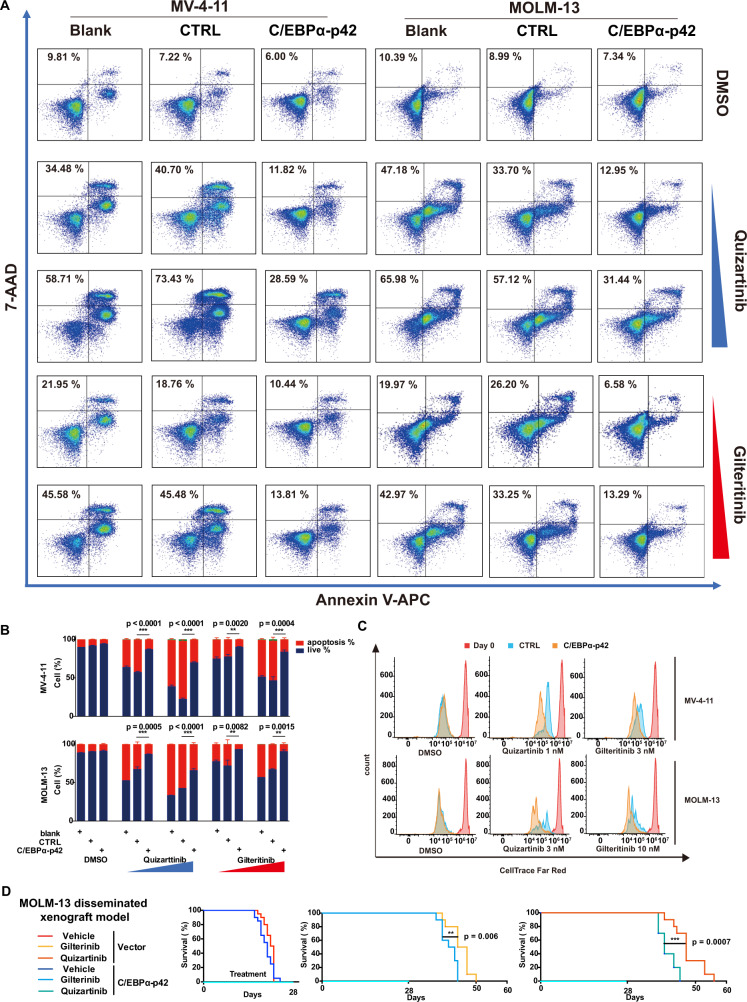
Overexpression of C/EBPα-p42 reduces the sensitivity of *FLT3 ITD* AML cells to FLT3i in vivo. **A** Apoptosis of MV-4-11 and MOLM-13 cells with or without C/EBPα-p42 overexpression treated with DMSO, quizartinib (1, 3 nM) or gilteritinib (3, 10 nM) in MV-4-11 cells and quizartinib (3, 10 nM) or gilteritinib (10, 30 nM) in MOLM-13 cells for 48 h. **B** Quantification of the apoptosis changes in (**A**). *n* = 3 independent experiments. Data are Means ± SEM. Significance was analyzed by equal variance two-tailed *t* test. *P < 0.05, **P < 0.01, *** *P* < 0.001. **C** CellTrace™ Far Red staining of MV-4-11 and MOLM-13 cells with or without C/EBPα-p42 overexpression treated with DMSO, quizartinib or gilteritinib for 4 days. **D** Survival curve of B-NDG mice xenografted with MOLM-13 cells with or without C/EBPα-p42 overexpression and treated with gilteritinib (30 mg/kg, *n* = 10 mice), quizartinib (9 mg/kg, *n* = 10 mice) or 22% β-CD as the vehicle (*n* = 20 mice). Significance was a*n*alyzed by logrank test. *P < 0.05, **P < 0.01, *** *P* < 0.001. Exact *p* values in Source Data file. Data for all graphs in Source Data file.

To determine whether C/EBPα activation leads to FLT3i resistance, we infected *FLT3-ITD* AML cell lines (MV-4-11 and MOLM-13) with lentivirus carrying constitutively active C/EBPα-p42 (Fig. 2A) to generate respective stable cell lines and assessed the transcriptome of these C/EBPα-p42-overexpressing cells. There was approximately 1.65- and 1.80-fold overexpression of C/EBPα-p42 in MV-4-11 and MOLM-13 cells, respectively (Supplementary Fig. 4A, B). C/EBPα-regulated target genes, including those in the ND and immune-related pathways, were significantly upregulated in both cell lines (Fig. 2B, C, Supplementary Data 3), consistent with the notion that the ND- and immune-related pathways could be regulated by C/EBPα. The unsupervised heatmaps could also clearly distinguish the C/EBPα-P42 overexpression and control groups in MV-4-11 and MOLM-13 cells (Supplementary Fig. 5A, B). Moreover, overexpression of C/EBPα-p42 reduced the extent of quizartinib and gilteritinib in suppressing the STAT5, AKT, and ERK pathways in MV-4-11 and MOLM-13 cells (Fig. 2D, E). Accordingly, overexpression of C/EBPα-p42 could reduce quizartinib and gilteritinib induced cell growth inhibition (Fig. 2F), cell apoptosis (Fig. 3A, B) and proliferation inhibition (Fig. 3C). Then, the effects of C/EBPα-p42 on AML progression and the response to FLT3i indisseminated MOLM-13 and MV-4-11 AML mouse models were evaluated. In the MOLM-13 model, C/EBPα-p42 overexpression significantly reduced the survival time of mice treated with quizartinib (9 mg/kg) and gilteritinib (30 mg/kg) (median survival 40 vs. 47 days; *P* = 0.0007; 42 vs. 45.5 days; *P* = 0.006) (Fig. 3D). C/EBPα-p42 overexpression significantly reduced the survival time of mice treated with quizartinib (3 mg/kg) and gilteritinib (3 mg/kg) (median survival 58.0 vs. 89.0 days; *P* = 0.0001; 48.0 vs. 63.0 days; *P* = 0.004) in the MV-4-11 model (Supplementary Fig. 6A). Both results indicated that C/EBPa-p42 overexpression lowers FLT3i sensitivity in vivo.

### Inhibition of C/EBPα synergizes with FLT3i

**Fig. 4:**
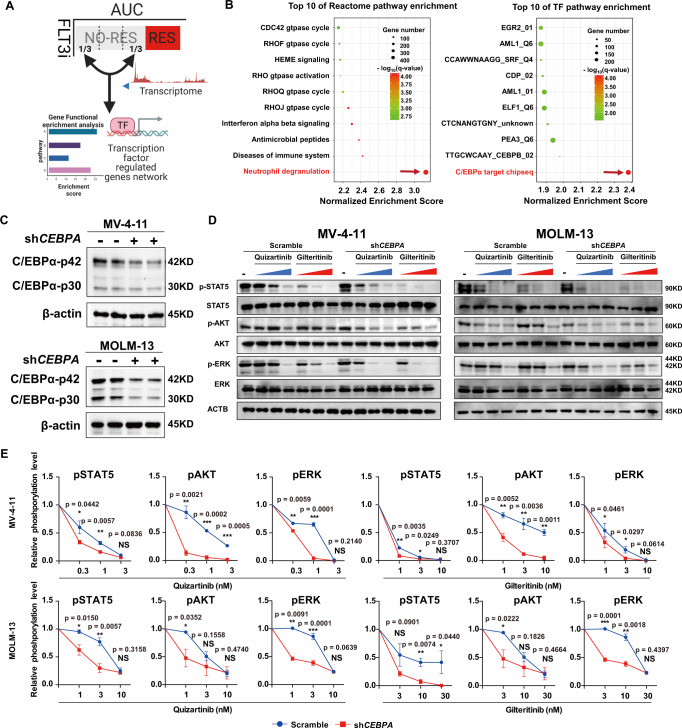
*CEBPA* knockdown enhances the FLT3i induced signal pathway downregualtion. **A** Schematic representation of RNA-seq analysis in the non-resistant FLT3i group. **B** Transcriptional features were enriched by GSEA. **C** *CEBPA* knockdown in MV-4-11 and MOLM-13 cell lines. **D** Changes in STAT5, AKT and ERK phosphorylation in MV-4-11 and MOLM-13 cells with or without *CEBPA* knockdown treated with quizatinib (0.3, 1, or 3 nM) or gilteritinib (1, 3 or 10 nM) in MV-4-11 and quizatinib (1, 3, or 10 nM) or gilteritinib (3, 10, or 30 nM) in MOLM-13 for 2 h. **E** Quantification of the phosphorylation changes in (**D**). *n* = 3 independent experiments. Data are Means ± SEM. Significance was analyzed by equal variance two-tailed *t* test.*P < 0.05, **P < 0.01, ***P < 0.001. Exact *p* values in Source Data file. Data for all graphs in Source Data file.

**Fig. 5:**
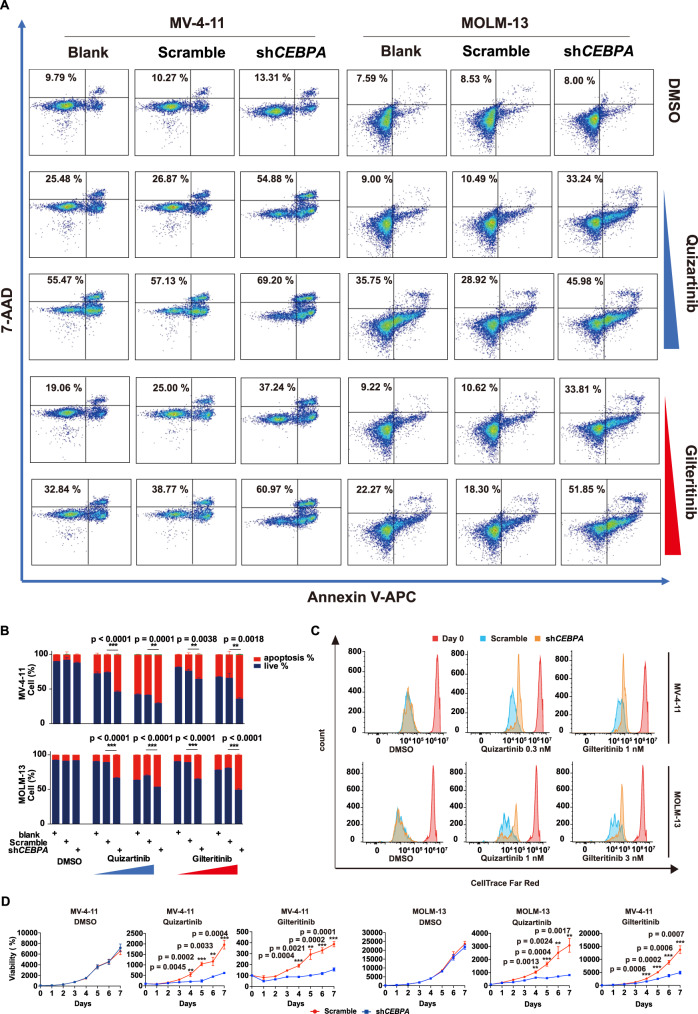
*CEBPA* knockdown enhances the sensitivity of *FLT3-ITD* AML to FLT3i. **A** Cell apoptosis of MV-4-11 and MOLM-13 cells with and without *CEBPA* knockdown treated with DMSO, quizartinib (0.3, 1 nM) or gilteritinib (1, 3 nM) in MV-4-11 and quizartinib (1, 3 nM) or gilteritinib (3, 10 nM) in MOLM-13 for 48 h. **B** Quantification of the apoptosis changes in (**A**). *n* = 3 independent experiments. Data are means ± SEM. Significance was analyzed by equal variance two-tailed *t* test. *P < 0.05, **P < 0.01, ***P < 0.001. **C** CellTrace™ FAR RED staining of MV-4-11 and MOLM-13 cells with and without *CEBPA* knockdown treated with DMSO, quizartinib or gilteritinib for 4 days. **D** The cell growth of MV-4-11 and MOLM-13 cells with or without *CEBPA* knockdown treated with quizartinib or gilteritinib. *n* = 3 independent experiments. Data are means ± SEM. Significance was analyzed by equal variance two-tailed *t* test. *P < 0.05, **P < 0.01, ***P < 0.001. Exact *p* values in Source Data file. Data for all graphs in Source Data file.

**Fig. 6:**
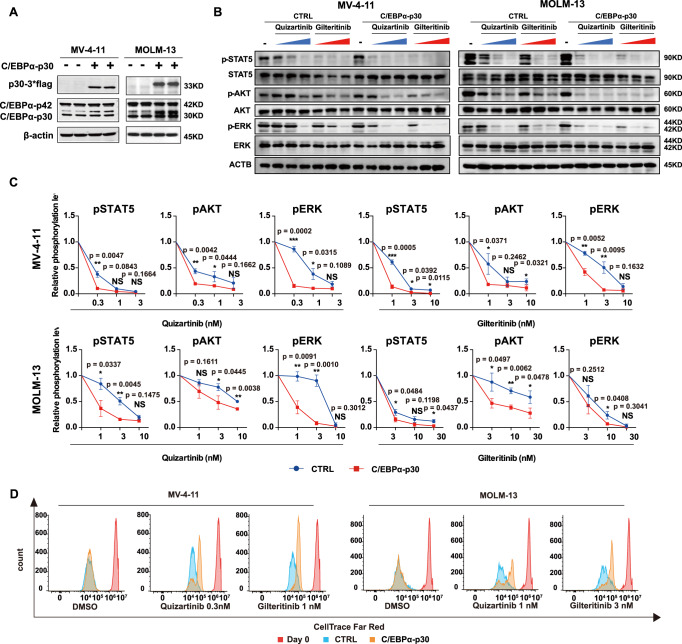
Overexpression of C/EBPα-p30 enhances the sensitivity of *FLT3-ITD* AML to FLT3i in vitro. **A** C/EBPα-p30 was overexpressed in MV-4-11 and MOLM-13 cells. **B** Changes in STAT5, AKT and ERK phosphorylation in MV-4-11 and MOLM-13 cells with or without C/EBPα-p30 overexpression treated with the same treatment in Fig. 4D. **C** Quantification of the phosphorylation changes in (**B**). *n* = 3 independent experiments. Data are means ± SEM. Significance was analyzed by equal variance two-tailed *t* test. *P < 0.05, **P < 0.01, ***P < 0.001. **D** CellTrace™ FAR RED staining of MV-4-11 and MOLM-13 cells with or without C/EBPα-p30 treated with DMSO, quizartinib or gilteritinib for 4 days. Exact p-values in Source Data file. Data for all graphs in Source Data file.

**Fig. 7:**
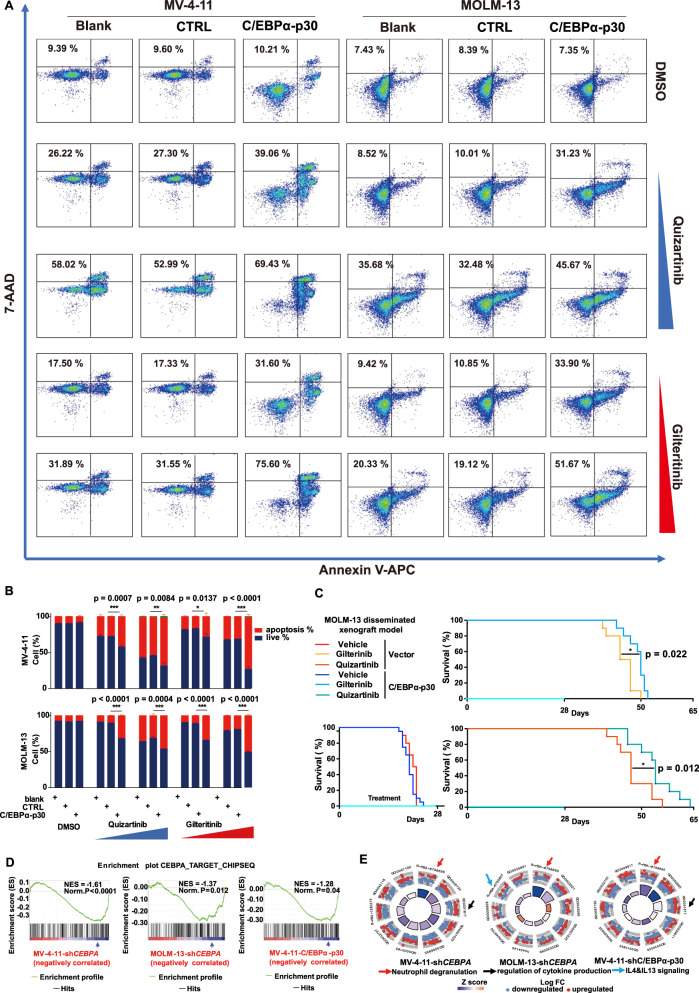
Overexpression of C/EBPα-p30 enhances the sensitivity of *FLT3-ITD* AML to FLT3i in vivo. **A** Cell apoptosis of MV-4-11 and MOLM-13 cells with and without C/EBPα-p30 treated with the same treatment in Fig. 5A. **B** Quantification of the apoptosis changes in (**A**). *n* = 3 independent experiments. Data are means ± SEM. Significance was analyzed by equal variance two-tailed *t* test. *P < 0.05, **P < 0.01, *** *P* < 0.001. **C** Survival of B-NDG mice xenografted with MOLM-13 with or without C/EBPα-p30 overexpression and treated with gilteritinib (30 mg/kg, *n* = 10 mice), quizartinib (9 mg/kg, *n* = 10 mice), or vehicle control *(n* = 20 mice). Significance was a*n*alyzed by *logrank* test. *P < 0.05, **P < 0.01, *** *P* < 0.001. **D** GSEA enrichment of C/EBPα target genes in *CEBPA* knockdown or C/EBPα-p30-overexpressing MV-4-11 or MOLM-13 cells. **E** Functional enrichment of the differential signalling in *CEBPA* knockdown or C/EBPα-p30-overexpressing MV-4-11 or MOLM-13 cells. Exact *p* values in Source Data file. Data for all graphs in Source Data file.

To further examine the relationship between C/EBPα activation and FLT3i sensitivity, we divided the samples of the top two-thirds (non-resistant group) into three subgroups based on drug sensitivity and compared the top one-third (MS or more sensitive subgroup) with the bottom one-third (LS or less sensitive subgroup) for transcriptional features, including pathways and TFs (Fig. 4A). We then used GSEA to interrogate the average rank for each gene to define an average-rank signature (Supplementary Data 4). The ND pathway and C/EBPα activation were still the most significantly enriched in the LS subgroup (Fig. 4B), indicating that inhibition of C/EBPα could enhance the response to FLT3i in the non-resistant group. To this end, we generated stable *CEBPA* knockdown (KD) MV-4-11 and MOLM-13 cell lines by lentivirus-mediated shRNA^20^ (Fig. 4C, Supplementary Fig. 7). *CEBPA* KD further enhanced FLT3i-induced inhibition of the STAT5, AKT, and ERK pathways (Fig. 4D, E) and significantly improved FLT3i-induced cell apoptosis (Fig. 5A, B), cell proliferation (Fig. 5C), and cell growth inhibition (Fig. 5D) in both stable cell lines. C/EBPα-p30, which retains the C-terminal domain but lacks part of the N-terminal transactivation domain of C/EBPα, can inhibit wild-type C/EBPα-p42 in a dominant-negative manner^21^. This prompted us to also investigate the effect of C/EBPα-p30 on the sensitivity of *FLT3-ITD* AML cells to FLT3i treatment. Similar to *CEBPA* KD, overexpression of C/EBPα-p30 significantly enhanced the FLT3i-induced inhibition of STAT5, AKT and ERK phosphorylation (Fig. 6A, B, C, Supplementary Fig. 4), cell proliferation (Fig. 6D), and cell apoptosis (Fig. 7A, B). We then examined the effect of C/EBPα-p30 overexpression on the responsiveness to FLT3i in disseminated MOLM-13 and MV-4-11 AML mouse models. We found that C/EBPα-p30 overexpression significantly extended the median survival time (quizartinib, 9 mg/kg, daily, 47 to 54 days, *P* = 0.022) (gilteritinib, 30 mg/kg, daily, 45.5 to 50 days; *P* = 0.012) compared to the vector (empty-LV) control group in the MOLM-13 model (Fig. 7C). In the MV-4-11 model, C/EBPα-p30 overexpression significantly extended the median survival time (quizartinib, 3 mg/kg, daily, 95.0 to 76.0 days, *P* = 0.002, and gilteritinib, 3 mg/kg, daily, 60.5 to 54.5 days, *P* = 0.025) compared to the vector control group (Supplementary Fig. 6B). Both KD of *CEBPA* and overexpression of C/EBPα-p30 significantly downregulated the expression of C/EBPα target genes (Fig. 7D), including the ND, IL-4 and IL-13 cytokine pathways, in both MV-4-11 and MOLM-13 cells (Fig. 7E, Supplementary Data 5), suggesting an on-target effect. Taken together, these results support that inhibition of C/EBPα enhances FLT3i sensitivity in both in vitro and in vivo models.

To pursue whether C/EBPα and FLT3 inhibitor resistance are specific, more experiments were performed. *FLT3* WT HL-60 cells were overexpressed with C/EBPα-p42 and sh*CEBPA*, and then, the effects of FLT3i on such engineered cells were evaluated with CTG or apoptosis assays. C/EBPα-p42 and *shCEBPA* overexpression showed less effect on the sensitivity of FLT3i (Supplementary Fig. 8). The combination of guanfacine and FLT3i showed no synergistic effect in *FLT3* WT AML cells (Supplementary Fig. 9). Then, other AML clinical drugs, such as cytarabine and flavopiridol, were used to test their sensitivity in MV-4-11 and MOLM-13 cells overexpressing C/EBPα-p42, p30 and *shCEBPA* (Supplementary Fig. 10). The pan-CDK inhibitor flavopiridol-induced cell apoptosis was enhanced in C/EBPα-p42 cells but suppressed in C/EBPα-p30 and *shCEBPA* cells. There were no clear differences among cytarabine-induced apoptosis in C/EBPα-p30, *shCEBPA*, C/EBPα-p42 and control cells. While, FLT3i-induced cell apoptosis was enhanced in C/EBPα-p30 and *shCEBPA* cells but suppressed in C/EBPα-p42 cells compared to control *FLT3-ITD* cells (Figs. 2F–I,  5A–D,  6C, D,  7A, B). Therefore, we could draw the conclusion cautiously that C/EBPα-induced drug resistance is not broad-spectrum but selective and specific for FLT3i in *FLT3-ITD* AML cells.

### Repurposed drugs targeting C/EBPα enhance FLT3i efficacy

**Fig. 8:**
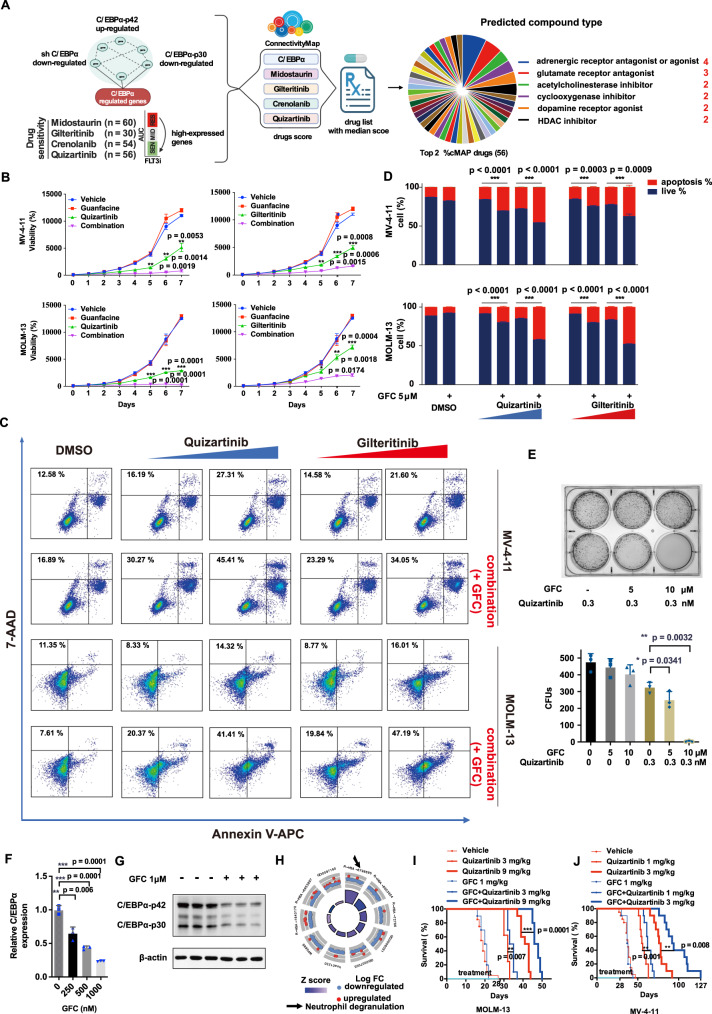
Repurposing drugs targeting C/EBPα enhances the antileukaemic efficacy of FLT3i in vitro and in vivo. **A** The drug repurposing discovery process of targeting C/EBPα via cMAP analysis. A score ≤ −90 indicated significant downregulation. Drugs targeting adrenergic receptors accounted for the largest proportion (7%, 4 cases) of the top 2% lowest-scoring drugs (all ≤ −90). **B** The cell growth curve of MV-4-11 and MOLM-13 cells treated with quizartinib or gilteritinib in the absence or presence of guanfacine (GFC). *n* = 3 independent experiments. Data are Means ± SEM. Significance was analyzed by equal variance two-tailed *t* test. *P < 0.05, **P < 0.01, ***P < 0.001. **C** Cell apoptosis of MV-4-11 and MOLM-13 cells with and without GFC (5 μM) treated with DMSO, quizartinib (0.1, 0.3 nM) or gilteritinib (0.3, 1 nM) in MV-4-11, and quizartinib (0.3, 1 nM) or gilteritinib (1, 3 nM) in MOLM-13 for 48 h. **D** Quantification of the apoptosis changes in (**C**). *n* = 3 independent experiments. Data are means ± SEM. Significance was analyzed by equal variance two-tailed *t* test. *P < 0.05, **P < 0.01, ***P < 0.001. **E** Representative clone formation of MV-4-11 cells treated with GFC, quizartinib, or their combination. Data are Means ± SEM. *n* = 3 independent experiments. Significance was analyzed by equal variance two-tailed *t* test. *P < 0.05, **P < 0.01, ***P < 0.001. **F** The effect of GFC treatment on the expression of the *CEBPA* gene in MV-4-11 cells, as detected by qPCR. *n* = 3 independent experiments. Significance was analyzed by equal variance two-tailed *t* test. *P < 0.05, **P < 0.01, ***P < 0.001. **G** The effect of GFC treatment on the expression of *CEBPA* in MV-4-11 cells, as detected by western blotting. *n* = 3 independent biological replicates. **H** Functional enrichment analysis of the differential signalling pathways in GFC-treated MV-4-11 cells. **I** Survival curve of B-NDG mouse disseminated xenografted MOLM-13 cells treated with vehicle (22% Beta-CD, *n* = 20 mice), quizartinib (3 or 9 mg/kg, *n* = 10 mice), GFC (1 mg/kg, *n* = 10 mice), or the combination (*n* = 10 mice). **J** Survival curve of B-NDG mouse disseminated xenografted MV-4-11 cells treated with vehicle (22% Beta-CD, *n* = 20 mice), quizartinib (1 or 3 mg/kg, *n* = 10 mice), GFC (1 mg/kg, *n* = 10 mice), or the combination (*n* = 10 mice). Significance was analyzed by *logrank* test. *P < 0.05, **P < 0.01, ***P < 0.001. Exact *p* values in Source Data file. Data for all graphs in Source Data file.

Considering that C/EBPα activation enhances FLT3i resistance (Figs. 2 and 3) and that inactivation of C/EBPα enhances FLT3i sensitivity (Figs. 4–7), we reason that targeting C/EBPα and its regulated gene network may be a viable approach for enhancing FLT3i sensitivity and overcoming FLT3i PR. Connectivity Map (cMAP) is a collection of genome-wide gene expression readouts of cell lines treated with more than 2000 drug-like chemicals, which allows identification of molecules based on expression profile similarity^22,23^. We input into the cMAP database the common genes that were upregulated in C/EBPα-overexpressing cells and downregulated in *CEBPA* KD cells and C/EBPα-p30-overexpressing cells (Supplementary Data 6); then, the FLT3i resistance-associated gene sets were upregulated by C/EBPα (Supplementary Data 6). This generated five lists of drugs targeting the common genes and the four FLT3i-associated C/EBPα-upregulated genes, with connectivity scores (see methods for details) representing reversing (low score) or mimicking (high score) expression signatures with the input genes (Fig. 8A). The summary drug score was calculated with median statistical analysis (Supplementary Data 7). Four drugs targeting adrenergic receptors (ARs) appeared in the top 2% of drugs that could downregulate the input genes (Supplementary table 1). Among them, three drugs (guanfacine, esmolol, and practolol) were approved for the treatment of hypertension, attention deficit hyperactivity disorder (ADHD) and other diseases. Guanfacine, esmolol, and practolol did not show single-agent toxicity even at 10 μM in the MV-4-11 and MOLM-13 cell lines but could significantly enhance FLT3i-induced cell growth inhibition (Fig. 8B, Supplementary Fig. 11) when compared with treatment with FLT3i alone. Guanfacine, the only oral drug among the three, was chosen for further study due to its high compliance and safety. We found that guanfacine significantly enhanced FLT3i-induced cell apoptosis (Fig. 8C, D). Then, we examined whether guanfacine could promote FLT3i-induced cell death upon prolonged exposure by treating MV-4-11 cells with quizartinib alone or combined with guanfacine for 14 days and found that clone formation decreased significantly in the combination treatment (Fig. 8E). Guanfacine treatment led to reduced C/EBPα expression at both the mRNA (Fig. 8F) and protein levels (Fig. 8G) and consequently decreased the ND pathway (Fig. 8H, Supplementary Data 8).

To determine whether guanfacine could enhance FLT3i sensitivity in vivo, we established a xenograft tumour model by subcutaneous inoculation of MV-4-11 cells into nu/nu mice and treated the animals with vehicle, the single-agent guanfacine, quizartinib, or the combination when the tumours reached 100-300 mm^3^. Guanfacine (1 mg/kg, daily) alone caused no significant changes in tumour growth, while quizartinib (0.5 mg/kg, daily) alone slowed tumour growth compared to the vehicle group. Importantly, the combination of guanfacine and quizartinib significantly reduced the tumour burden compared with quizartinib (*P* = 0.0286) or vehicle treatment (*P* = 0.0139) (Supplementary Fig. 12). A similar effect of combination treatment with guanfacine and quizartinib was observed in the disseminated MOLM-13 and MV-4-11 AML mouse models (Fig. 8I, J). Guanfacine alone had no effect on the mouse survival time, while quizartinib alone prolonged the mouse survival time in a dose-dependent manner in both models. Strikingly, the combination treatment further substantially prolonged overall survival compared with single-agent quizartinib at doses of 3 and 9 mg/kg in the MOLM-13 model (median survival time 34 vs. 32 days, *P* = 0.007; 47 vs. 42 days, *P* = 0.0001) (Fig. 8I) and compared with single-agent quizartinib at doses of 1 and 3 mg/kg in the MV-4-11 model (median survival time 62.0 vs. 55.5 days, *P* = 0.008; 93 vs. 77 days, *P* = 0.001) (Fig. 8J).

Collectively, the findings that guanfacine could enhance FLT3i sensitivity in in vitro and in vivo models highlight that the gene networks regulated by C/EBPα activation could be successfully targeted by repurposing existing drugs with apparent efficacy.

### Further validation of C/EBPα as a targetable mechanism for enhancing FLT3i sensitivity in independent patient samples

**Fig. 9:**
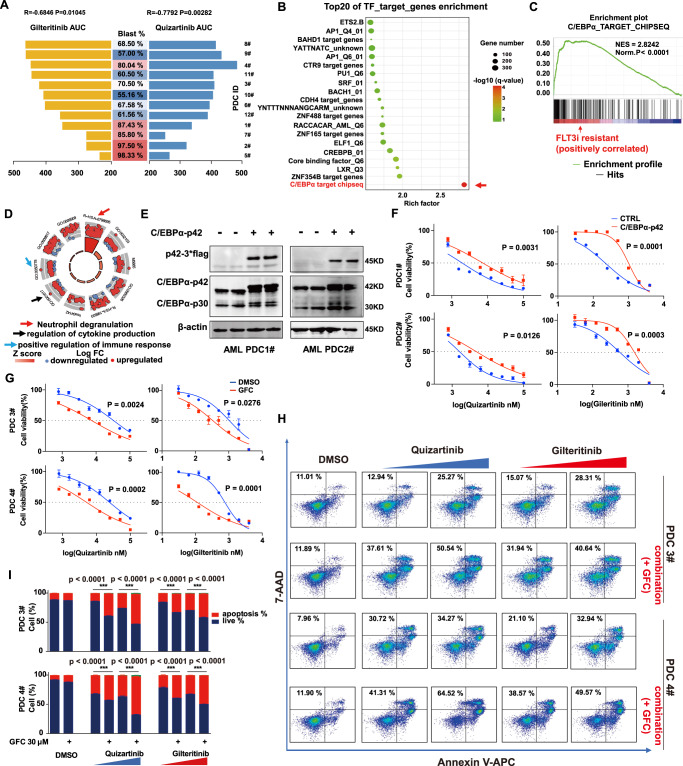
Validation of C/EBPα as a targetable mechanism for enhancing FLT3i sensitivity in independent patient samples. **A** The correlation of the AUC value of the twelve *FLT3-ITD* AML PDC samples to gilteritinib and quizartinib in vitro and the blasts (%) in the bone marrow. The correlation and significance were calculated by the Spearman method. **B** Transcription factor enrichment analysis in the FLT3i-resistant group. **C** GSEA plots (included p-value and enrichment score) of the transcription factor C/EBPα-regulated genes enriched in the FLT3i-resistant group. **D** Functional enrichment analysis of the differential signalling pathways in the FLT3i-resistant group. **E** C/EBPα-p42 was overexpressed in two *FLT3-ITD* AML PDCs, as confirmed by western blotting. **F** The viabilities of PDCs with and without C/EBPα-p42 overexpression at the indicated concentrations of quizartinib and gilteritinib. *n* = 3 independent experiments. **G** The viabilities of PDCs in the absence or presence of guanfacine (100 μM) and at the indicated concentrations of quizartinib and gilteritinib. *n* = 3 independent experiments. Significance was analyzed by equal variance two-tailed *t* test. *P < 0.05, **P < 0.01, ***P < 0.001. **H** Cell apoptosis of PDC cells with and without GFC (30 μM) treated with DMSO, quizartinib (300, 1000 nM) or gilteritinib (150, 500 nM) in PDC3# and quizartinib (1000, 3000 nM) or gilteritinib (150, 500 nM) in PDC4# for 48 h. **I** Quantification of the apoptosis changes in (**H**). *n* = 3 independent experiments. Data are means ± SEM. Significance was analyzed by equal variance two-tailed *t* test. *P < 0.05, **P < 0.01, ***P < 0.001. Exact *p* values in Source Data file. Data for all graphs in Source Data file.

Twelve *FLT3-ITD* AML patient-derived cells (PDCs) were confirmed by fluorescent PCR and capillary electrophoresis. They were then sequenced and analyzed for their sensitivity to quizartinib and gilteritinib in vitro. None of them contained *MLL* fusions. Consistent with the above findings (Fig. 1A), samples with high blasts (%) were more sensitive to gilteritinib and quizatinib (Fig. 9A, Supplementary Data 9). WES data showed few overall somatic mutations, except for *NPM1* mutations, which accounted for ~33% (4/12) (Supplementary Fig. 13A). Patients with *NPM1* mutations showed comparable sensitivity to FLT3i (gilteritinib, *P* = 0.11; quizartinib, *P* = 0.02) (Supplementary Fig. 13B). We analyzed gene expression differences between the FLT3i-sensitive group (*n* = 5, with AUC < 400 in both quizartinib and gilteritinib treatment) and the FLT3i-resistant group (*n* = 7). Similar to the findings using the Vizome database (Supplementary Fig. 6A), the C/EBPα target gene set was most significantly upregulated in the drug-resistant group among all transcription factor pathways (Fig. 9B-C, Supplementary Data 10). In addition, ND and immune-related pathways were upregulated in the resistant group (Fig. 9D). Next, we determined whether genetic activation of C/EBPα or pharmacological inhibition of C/EBPα in PDC could alter the response to FLT3i. We infected two sensitive FLT3-ITD AML PDCs (1# and 2#) with lentivirus carrying C/EBPα-p42 (Fig. 9E), which induced a >2-fold increase in the IC_50_ of quizartinib and gilteritinib (Fig. 9F, Supplementary table 2). We found synergistic effects when combining FLT3i and guanfacine in treating FLT3i-resistant PDCs (3# and 4#), where supplementation with guanfacine induced a >3-fold decrease in the IC_50_ of quizartinib and gilteritinib in PDC samples and significantly enhanced FLT3i-induced cell apoptosis (Fig. 9H, I), showing a high synergistic effect (Supplementary Fig. 14, Supplementary table 3). Together, these results provide further validation that C/EBPα is a viable target in reversing FLT3i resistance and support clinical studies to examine combining FLT3i and guanfacine in the treatment of FLT3i PR patients.

## Discussion

Acquired resistance to FLT3i is usually driven by the emergence of secondary resistance gene mutations, including FLT3 activation loops or gatekeeper residues, or parallel prosurvival signal emergency mutations in genes of the PI3K/AKT, JAK, RAS/MEK/MAPK pathways^24–27^. Notably, the acquired resistance mutation patterns responding to type I and type II FLT3i are significantly different^14^, and even some secondary mutations, such as *NRAS*, are associated with only late-stage resistance in AML cells^5^. However, to our knowledge, only one study has reported rare *CCND3* gene mutations related to primary drug resistance^28^. Adaptive signalling pathway activation is another mechanism that responds more quickly to FLT3i resistance and does not depend on gene mutations. The signals that mediate acquired resistance to FLT3i mainly include oncogenic kinases, such as PIM-2, AXL, Aurora B, and IRAK1/4, and related innate immune or inflammatory pathways^29–31^. However, no signalling pathways related to primary drug resistance have been identified.

Here, we report the unbiased findings on the PR features to FLT3i in clinical characteristics, gene mutations and gene expression levels using multiple datasets of *FLT3-ITD* AML patients. Not surprisingly, we did not find any gene mutations that resulted in PR of FLT3i but instead *NPM1* mutations that rendered the patients more sensitive to FLT3i, consistent with previous reports^32,33^. Our analyzes indicate that identifying and targeting specific gene mutations for the treatment of FLT3i PR may not be an effective approach and suggest targeting aberrant signalling pathways to reverse PR. As such, we focused on identifying the TFs and their associated signalling pathways responsible for the observed changes in gene expression. Targeting TFs and/or their signalling pathways, rather than individual mutated genes, is expected to be more effective and may potentially prevent cancer cells from developing resistance^34^. We discovered C/EBPα and its activation as the only TF signature for common PR to type I and type II FLT3i. Patients with altered C/EBPα activity and those with NPM1 mutations share clinical blast features and FLT3i sensitivity. *NPM1* mutations, which result in abnormal cytoplasmic localization, repress monocyte and granulocyte terminal differentiation by disrupting PU.1-C/EBPα complex formation and enhancing FLT3i sensitivity^35^. AML patients with lower C/EBPα activity appear to have a higher PB blast cell count^36^ and higher sensitivity to FLT3i (Figs. 1B and 5A). These findings indicate that the C/EBPα-regulated gene network may be used as a companion biomarker, for stratification of *FLT3-ITD* patients in particular, and potentially for AML patients in general in their treatment.

Multiple lines of evidence support that C/EBPα activation is also a targetable mechanism to overcome FLT3i PR and enhance its efficacy. We show, in a gain-of-function study, that C/EBPα activation could lower the FLT3i response in FLT3i-sensitive cellular and animal models (Fig. 2). Second, we identified that C/EBPα activation remained the top pathway in the less sensitive subgroup when we further subdivided the non-resistant groups and performed pathway analysis in the subgroups (Fig. 4A, B). Third, we performed shRNA KD of C/EBPα and overexpression of the dominant-negative C/EBPα-p30 to reduce C/EBPα activity and demonstrated that both approaches could enhance the FLT3i response in non-resistant cells in in vitro and in vivo studies (Fig. 4C–E, Figs. 5–7). Both MOLM-13 and MV-4-11 contain *MLL* fusions. As the *FLT3-ITD* co-occurrence mutation with *MLL* fusion is rare in the clinic, a series of experiments in a number of primary AML PDC samples were performed to support the findings in cell lines (Fig. 9). Finally, we verified the importance of C/EBPα and confirmed C/EBPα activation as the most altered pathway in an independent cohort of twelve *FLT3-ITD* patients (Fig. 9A–D). Consistently, overexpression of C/EBPα resulted in a dramatic reduction in the FLT3i response in two FLT3i-sensitive PDC samples (Fig. 9E, F, Supplementary table 2). These findings not only provide the urgently needed targetable mechanism for FLT3i PR but also offer a potential strategy to combine downregulation of C/EBPα activity and FLT3i to achieve better treatment efficacy.

At present, no available molecules can directly inhibit C/EBPα activity. Drug repurposing is an attractive strategy for exploiting other indications for a drug beyond its original use^37^, and cMAP containing integrated cellular signatures based on network pharmacology has been used to approach drug repurposing in AML^38^. Our cMAP analysis revealed that several distinct pathway modulators could mimic C/EBPα in affecting the identified gene network changes (Fig. 8A). Given that AR signalling is the top pathway and several identified compounds have been approved by the FDA for clinical indications, we decided to focus on AR agonists for the initial proof-of-concept study. Guanfacine is a selective α2A-AR agonist that has been approved for the treatment of moderate to severe hypertension and ADHD. Impressively, guanfacine enhanced FLT3i sensitivity with robust synergy across cell lines and primary AML samples in vitro and in vivo (Figs. 8B–E, 4I, J and 5G–I). We have confirmed that guanfacine downregulates *CEBPA* gene expression, which in turn results in a similar gene network signature as *CEBPA* KD in the cMAP analysis, although we cannot rule out the possibility that guanfacine could affect the gene network signature independent of C/EBPα (Fig. 8E–G). Moreover, the combined effect on C/EBPα activity and its regulated gene network signature induced by guanfacine likely inhibits the ND pathway, which may account for its synergistic effects with FLT3i (Fig. 8E–G). Mechanistically, α2A-AR agonists could downregulate PKC-mediated phosphorylation of CREB^39,40^, which is a positive regulator of the C/EBP family^41^, thus reducing C/EBPα activity under AR activation. Consistent with this model, we observed similar synergistic effects when practolol and esmolol, two other approved drugs targeting AR, were combined with FLT3i for the treatment (Supplementary Fig. 11A, B). Of note, the dosage of 1 mg/day guanfacine used in mice is comparable to the clinical dosage in humans, lending confidence to the clinical translation of this drug combination.

In this study, we also examined the involvement of C/EBPα activation in acquired or adaptive resistance to FLT3i. This notion is supported by the fact that treatment with quizartinib for 12 h or gilteritinib for 48 h could induce *CEBPA* gene expression and activate the C/EBPα-regulated gene network, including the ND pathway^29^ (Supplementary Fig. 15). Notably, overexpression of C/EBPα activates the innate immune pathway, which contributes to acquired resistance to FLT3i^29^. Moreover, *RAS* mutations contribute to acquired resistance, in addition to FLT3i PR^14^. Although data analysis is limited by the small number of *FLT3-ITD* patient samples with *RAS* mutations in the Vizome database, we did observe significant enrichment of known C/EBPα targets in *RAS* mutant AML samples, including ND and innate immune pathways (Supplementary Fig. 16), indicating that *RAS* mutation-mediated FLT3i resistance may be associated with C/EBPα activation and benefit from combination treatment of guanfacine and FLT3i.

C/EBPα, a member of the basic region leucine zipper transcription factor family^42^, regulates myeloid development and promotes granulocyte and monocyte differentiation^43^. The effects on granulocytic differentiation have also been evaluated by detecting the differentiation indicator of CD117 or CD11b via FACS^44^, where C/EBPα-p42 overexpression could improve MV-4-11 and PDC cell differentiation (Supplementary Fig. 17). Furthermore, we found that C/EBPα-p42 overexpression induces cell differentiation in MOLM-13, MV-4-11, and HL-60 cells according to Wright-Giemsa staining and Q-PCR analysis (Supplementary Fig. 18).

Loss-of-function mutations of *CEBPA* contribute to ~10% of AML development, consistent with a tumour suppressor role^45–48^. In contrast, deletion of *CEBPA* blocks normal granulocyte formation and prevents initiation of AML^49,50^. Overexpression of wild-type C/EBPα has no effect on cell proliferation in K562 leukaemia cells^51^ but decreases the growth rate by 2.7-fold in U937 lymphoma cells^52^. In the clinic, both *FLT3-ITD* and *CEBPΑ* mutations were relatively mutually exclusive, with 5-10% and 1% *FLT3* mutations in *CEBPΑsm* (single mutation) and *CEBPΑdm* (double mutation) patients, respectively^53–55^. The outcome of *CEBPΑsm* patients is drastically reduced by the additional presence of *FLT3-ITD*^53–56^ or is not affected by *FLT3* co-occurrence mutations^57^. FLT3 may be a direct downstream effector of C/EBPα. *CEBPΑdm* patients have lower *FLT3* expression, and shRNA silencing *CEBPA* results in *FLT3* downregulation; monoallelic *CEBPΑ* mutation could not induce significant change in *FLT3* expression, possibly due to the presence of the *CEBPΑ* wild-type^58^. Overexpression of C/EBPα-p42, -p30, or KD had less effect on the three important FLT3 downstream pathways of STAT5, AKT, and ERK (Supplementary Fig. 19). Accordingly, C/EBPα-p42 or C/EBPα-p30 overexpression had no obvious effect on MOLM-13 and MV-4-11 cell growth in vitro (Figs. 2I and 5D) and less effect on MOLM-13 in vivo (Figs. 3D and 7C), but significantly exacerbated or impeded MV-4-11 AML progression in vivo (Supplementary Fig. 6A, B). Nevertheless, FLT3-ITD accelerates leukaemia development in the *CEBPAdm* AML mouse model^59,60^. Such inconsistent effects of *CEBPA* in different AML in vivo models indicated that the role and mechanistic basis for C/EBPα in AML, especially *FLT3-ITD* AML, are not completely understood.

Mechanistically, activating FLT3 mutations could inhibit C/EBPα activity via ERK1/2-mediated phosphorylation^61^. Consistently, the activity of C/EBPα in *FLT3-ITD* AML was lower than wt AML in Vizome (Supplementary Fig. 20A). However, the activity of C/EBPα between FLT3i-resistant *FLT3-ITD* AML and WT AML samples showed less of a difference (Supplementary Fig. 20B). Therefore, theoretically, *FLT3-ITD* cells should have lower C/EBPα activity and show sensitivity to FLT3i. Our results indicated that C/EBPα activity was regulated not only by FLT3-ITD via phosphorylation but also by other mechanisms that we need to further explore. At present, there are no reports on the different clinical responses to FLT3i in *C/EBPA* WT, *CEBPAsm*, and *CEBPAdm* AML patients. This will be very interesting and helpful for understanding the role of both *FLT3* and *CEBPΑ* aberrations in leukaemia development using the clinical outcomes of AML patients carrying *FLT3* and *CEBPΑdm* co-occurrence mutations treated with FLT3i therapies. Future studies are required to address the many remaining questions, for example, the specific role of C/EBPα in AML, the activation status of C/EBPα in FLT3-mutant AML, and the C/EBPα activation mechanism.

In summary, we have identified and validated C/EBPα activation as the key PR mechanism for FLT3i in *FLT3-ITD* AML and further determined that combining existing drugs targeting C/EBPα and its regulated gene network with FLT3i can overcome PR and enhance the efficacy of FLT3i. We propose future clinical studies to assess combination therapy of FLT3i and C/EBPα inactivation as a next-generation AML treatment strategy.

## Methods

### Patient samples and clinical data

Samples were obtained with written and informed consent from all patients according to the Declaration of Helsinki. The study protocol was approved by the IRB (Institutional Review Board) of Changhai Hospital. The content and conclusions of this study are not related to gender and age. Mononuclear cells were isolated by Ficoll gradient centrifugation from freshly obtained bone marrow aspirates. Cell pellets were snap-frozen in liquid nitrogen for subsequent DNA isolation, freshly pelleted cells were lysed immediately in RNAiso (TAKARA) for subsequent RNA isolation, and freshly isolated mononuclear cells were used for an in vitro drug sensitivity assay within 24 h (described in the in vitro drug sensitivity assay). Epithelial cells were collected from the patient’s mouth, and genomic DNA was isolated as matched normal controls for WES (Qiagen). Clinical, prognostic, genetic, cytogenetic and pathologic laboratory data, as well as treatment and outcome data, were manually curated from the electronic medical records of the patient.

### B-NDG mouse disseminated xenograft acute leukaemia model

Female mice (6–8 weeks old) were purchased from Biocytogen Jiangsu Co., Ltd China, housed under specific pathogen-free (SPF) conditions with a 12 h light/dark cycle at 22 °C and controlled humidity (60 ± 10%). Animal use procedures were approved by the Committee for Laboratory Animal Research guidelines of the Shanghai Research Center for Model Organisms (IACUC approval 2021-12-LJ-124). Mice were inoculated intravenously with 1 × 10^7^ MV-4-11 cells or 2 × 10^5^ MOLM-13 cells via the tail vein. Mice were treated 5 days post engraftment with drugs or vehicle (22% beta cyclodextrin, 22% Beta-CD) for 4 weeks. Survival was monitored daily. Animals were humanely killed when they became visibly ill, in accordance with Institutional Animal Care and Use Committee (IACUC) Ethical Protocols. The maximum permitted weight loss is 15%. Significance was analyzed by *logrank* test. *P < 0.05, **P < 0.01, ***P < 0.001.

### Nu/Nu mouse subcutaneous xenograft model evaluation

Female nu/nu mice (6–8 weeks old) were purchased from Beijing Vital River Laboratory Animal Technology Co., Ltd., housed under SPF conditions with a 12 h light/dark cycle at 22 °C. Animal use procedures were approved by the Committee for Laboratory Animal Research guidelines of the Shanghai Research Center for Model Organisms (IACUC approval 2021-12-LJ-125). Mice were implanted subcutaneously in the right flank with 5 × 10^6^ MV-4-11 cells. Cells were resuspended in serum-free growth medium and mixed 1:1 with Matrigel (cat. No: 354248; BD Bioscience). After tumours grew to 100 mm^3^ in volume, drugs or the vehicle (22% Beta-CD) was administered orally every day for 3 weeks. Tumour volume, as an indicator of tumour growth, was monitored twice every week using digital calliper measurements. Body weight was measured twice every week. The calculation formula of tumour volume was *V*_T_ = 1/2 × *a* × *b*^2^ (Note: a and b represent length and width, respectively). The maximal tumour size permitted by the committee is 2000 mm^3^, and we have confirmed that the maximal tumour size was not exceeded. The maximum permitted weight loss is 15%.

### Culture of AML PDCs in vitro

Mononuclear cells isolated from the patient’s bone marrow were cultured in X-VIVO medium with 10% FBS and cytokines (IL-3, 100 μg/ml; IL-6, 50 μg/ml; IL-11, 25 μg/ml; FLT3-L, 50 μg/ml; SCF, 100 μg/ml; TPO, 100 μg/ml; G-CSF, 100 μg/ml; EPO, 100 μg/ml; GM-CSF, 50 μg/ml) at 37 °C and 5% CO_2_.

### In vitro drug sensitivity assay

Primary AML cells (1 × 10^4^ cells/well) were seeded into 96-well plates (PerkinElmer Culture Plate-96, White Opaque 96-well Microplate) containing dose gradients of the indicated single agents or FLT3i combinations in triplicate and cultured for 3 days. Cell viability was measured by the CellTiter-Glo (CTG, G7573, Promega) assay. Cell viability was determined by comparing the luciferase count of drug-treated cells to that of untreated controls, which was set as 100%. IC_50_ values were calculated by a regression curve to fit the analysis using GraphPad Prism 6 software, and the area under the regression curve (AUC) was used as an indicator of drug sensitivity. Cell viability % in per group are presented as mean ± SEM. Significance was analyzed by equal variance two-tailed *t* test. *P < 0.05, **P < 0.01, ***P < 0.001. *P* value can be found in source data.

### Cell growth curve assay

MV-4-11 (ATCC, Manassas, VA, USA, 1500 cells/well) and MOLM-13 cells (JCRB Cell Bank, Japan, 500 cells/well) in the logarithmic growth phase were seeded in 96-well plates (PerkinElmer Culture Plate-96, White Opaque 96-well Microplate) with IMDM or 1640 medium containing 10% fetal bovine serum and 0.1% penicillin/streptomycin. Within 2 h after inoculation, MV-4-11 or MOLM-13 cells were treated with 0.3 or 1 nM quizartinib and 1 or 3 nM gilteritinib, respectively. At the same time, DMSO treatment was used as the control group. Cell viability was determined every day using the CTG. The statistical software was GraphPad Prism 6. Cell viability % in per group are presented as mean ± SEM. Significance was analyzed by equal variance two-tailed *t* test. *P < 0.05, **P < 0.01, ***P < 0.001. All cell lines were authenticated by STR profiling, tested for mycoplasma contamination.

### Apoptosis assay

Apoptosis was detected by an Annexin V-APC/7AAD Apoptosis Detection Kit (GA1023-KGA1026, Keygentec, Nanjing, China) followed by flow cytometry analysis. Briefly, cells were treated or not with the indicated drug concentration and time, washed with PBS, stained with Annexin V-APC/7AAD and detected using flow cytometry (CytoFLEX flow cytometer, Beckman Coulter, Inc.). The apoptosis rate of stained cells was counted in APC/PC5.5. FACS sequential gating strategies of apoptosis analysis was showed in Supplementary Fig. 21A. Cell % in per group are presented as mean ± SEM. Significance was analyzed by equal variance two-tailed t test. *P < 0.05, **P < 0.01, *** *P* < 0.001.

### Cell proliferation assay

Invitrogen CellTrace Cell Proliferation Kits (C34572, Invitrogen) were used to monitor the generation of proliferating cells by FACS. Briefly, cell proliferation was followed for 4 days using CellTrace™ Far Red reagent. Cells were harvested and stained with 1 μM Far Red reagent, cultured for 4 days with or without drug treatment at the indicated concentration, and then analyzed using flow cytometry (CytoFLEX flow cytometer, Beckman Coulter, Inc.) using 630-nm excitation and 660-nm emission filters (APC). FACS sequential gating strategies of proliferation analysis was showed in Supplementary Fig. 21B.

### Clone formation assay

MethoCult H4100 clone formation medium was mixed with IMDM medium and 10% fetal bovine serum, and then, the compound and MV-4-11 cells (1500 cell/ml) were added. A total of 1500 cells per well were seeded into a 6-well plate, cultured for 14 days in a carbon dioxide incubator (37 °C, 5% CO_2_), and then added to 200 μL of nitrotetrazolium blue (NBT, N5514-10TAB, Sigma-Aldrich) solution (1 mg/ml). After taking pictures of the stained clones, the number of clones formed was observed and counted. Clones are presented as mean values ± SEM. Significance was analyzed by equal variance two-tailed *t* test. *P < 0.05, **P < 0.01, *** *P* < 0.001.

### Western blotting

Cell lines or PDC cells were exposed to different concentrations of FLT3i, guanfacine or DMSO. After that, protein lysates were made by 4 × Laemmli Sample Buffer (161073, BIORAD, Richmond, USA). Protein samples were separated by SDS-polyacrylamide gel electrophoresis (BIO-RAD, Richmond, USA), transferred Nitrocellulose (NC) membranes. After blocking with 5% skim milk for 60 min at room temperature, NC membranes were incubated with the primary antibody at 4 °C overnight before being washed three times with TBST. Then NC membranes were incubated with secondary antibodies for 1 h at room temperature. The signals were detected with ECL (KF8001 or KF8003, Affinity, China) using BIO-RAD ChemiDoc Touch Imaging system. The detailed antibodies information could be found in Supplementary data 11. The experiment was repeated 3 times. Relative phosphorylation level in per group are presented as mean ± SEM. Significance was analyzed by equal variance two-tailed *t* test.*P < 0.05, **P < 0.01, ***P < 0.001.

### RT-qPCR

After reverse transcripting the RNA of the sample into cDNA, the primers of the target gene were designed to perform PCR amplification reaction. SYBR green dye was added to the reaction system. A fluorescent signal amplification curve was obtained with the accumulation of reaction products to quantify the template. The expression of genes was normalized to the geometric mean of the housekeeping gene ACTB to control the variability in expression levels and was analyzed using the 2^−ΔΔCt^ method. The gene relative expression is presented as mean ± SEM. Significance was analyzed by equal variance two-tailed *t* test. *P < 0.05, **P < 0.01, ***P < 0.001. The primer sequnce can be found in Supplementary data 11.

### Lentivirus packaging and production

The endotoxin-free lentiviral vector and its packaging original vector plasmids were co-transfected into 293T cells with HG transgene reagent. After 8 h, fresh medium was added to the cells, and the cells were cultured for 48 h. The cell supernatant rich in lentivirus particles was concentrated to obtain high-titre lentivirus. Gene sequences can be found in Supplementary Data 11.

### Lentivirus infection of AML cell lines

In total, 4 × 10^4^ cells in 250 μL of medium were inoculated in a 24-well plate, and added with 10 μL of quantitative virus (MOI = 100) stock solution. Cells were centrifuged at 2000 rpm for 90 min at a flat angle and incubated for 2.5 h, and then, 250 μL of fresh medium was added per well. After 24 h, the cells were centrifuged, washed, and cultured with fresh medium. After 48 h, 1 μg/ml puromycin was added and screened for the stable expressing cells.

### Lentivirus infection of PDC

A total of 5 × 10^5^ cells in 500 μL of medium were inoculated in a 12-well plate, and added with 10 μL of quantitative virus (MOI = 10) stock solution. Cells were centrifuged at 2000 rpm for 90 min at a flat angle and incubated for 2.5 h, and then, 500 μL of fresh medium was added per well. After 24 h, the cells were centrifuged, washed, and cultured with fresh medium.

### Cell differentiation assay

The cell differentiation status was evaluated with the CD117/CD11b staining FACS assay or Wright-Giemsa staining. Briefly, 5 × 10^5^ MV-4-11 cells with or without C/EBPα-p42 expression were cultured with CD117 (c-Kit) monoclonal antibody for 30 min, washed with PBS, and then analyzed by flow cytometry (CytoFLEX flow cytometer, Beckman Coulter, Inc.) using a 488-nm excitation and 780-nm emission filter. 5 × 10^5^ PDC 2# cells with or without C/EBPα-p42 were cultured with a CD11b monoclonal antibody for 30 min, washed with PBS, and then analysedby flow cytometry (CytoFLEX flow cytometer, Beckman Coulter, Inc.) using 630-nm excitation and 660-nm emission filters. FACS sequential gating strategies of differentiation analysis was showed in Supplementary Fig. 21C. The detailed CD117 and CD11b antibodies information could be found in Supplementary data 11.

MOLM-13, MV-4-11, and HL-60 cells with or without C/EBPα-p42 expression were cultured and stained with a Fast Wright’s-Giemsa Stain Kit (60529ES01, Yeaseen, China) following the protocol in which a small drop of cells was smeared on a clean microscope slide, allowed to air dry, and then fixed with absolute methanol for 5 min. Slides were stained and flooded with working Wright Giemsa solution for 5 min, rinsed in distilled water, air dried at room temperature, and viewed and photographed under a 600× microscope (Olympus IX73).

### Detection of *FLT3-ITD* mutation by fluorescent PCR-capillary electrophoresis

Fluorescent PCR-capillary electrophoresis was used to detect whether there was *FLT3-ITD* mutation in bone marrow blood samples. Fluorescent primers were synthesized, and an ABI3730xl sequencer was used for capillary electrophoresis. GeneMapper 3.2 was used for data analysis. If the amplified peaks or double peaks were inconsistent with the size of 330 bp (WT product size), it was considered that there was an FLT3-ITD mutation in the sample. Sequences of primers can be found in Supplementary Data 11.

### WES data analysis

WES sequencing reads after exclusion of low-quality reads were mapped to the UCSC hg19 reference sequence with BWA. Removing and recalibrating PCR duplicates and detecting germline mutations were executed by GATK 4.0. Healthy human mutation databases (GenBank, ExAC, 1000G) and patient oral epithelial samples were defined as control samples, and somatic mutations were detected by comparison with the control samples.

### RNA-seq data analysis

Sequencing reads were aligned using hisat2 to the human reference sequence (UCSC hg19 assembly) after removal of adaptor contamination, polyA and polyC. Samples were used to compress and sort the aligned files, and gene expression values were quantitated with htseq-count. The RNA expression data of Fig. 1 were obtained from the Vizome database, and the differentially expressed genes were identified by the DESeq2 package in R software using twofold change (log2 (fold-change) ≥1 or ≤−1) and *P* < 0.05 (cut-off at 5% false discovery rate (FDR)) as the threshold. The differentially expressed genes were enriched using the online software Metascape (www.metascape.org). The enrichment algorithm was Fisher’s exact test, and the reference databases of pathways included KEGG, GO, Reactome databases.

### Gene set enrichment analysis

GSEA was performed using GSEA 4.1.0 software according to the running-sum statistic and the weighted Kolmogorov–Smirnov-like statistic (https://www.gsea-msigdb.org/gsea/datasets.jsp). Significance of GSEA results was determined by the two-sided permutation test, and *P* value was adjusted for multiple comparisons.

We prepared TXT files (gene expression matrix: RNAseq of MV-4-11 and MOLM-13, each sample contains three replicates), CLS files (grouping data, CTRL vs. *CEBPA-p42*, CTRL vs. *CEBPA-p30*, scramble vs. sh*CEBPA*), and GMT files (pathway database, transcription factor targets, https://www.gsea-msigdb.org/gsea/msigdb/collections.jsp) and input the series of documents into GSEA software (https://www.gsea-msigdb.org/gsea/datasets.jsp). The signal2noise score was selected to rank the degree of gene change. We enriched the pathway according to running-sum statistics and weighted Kolmogorov–Smirnov-like statistics. Finally, we ranked the pathways according to the normalized enrichment score and false discovery rate to find the significant changes in transcription factor target pathways between the experimental and control groups (CTRL vs. *CEBPA-p42*, CTRL vs. *CEBPA-p30*, scramble vs. sh*CEBPA*).

### The mining flowchart of repurposed drugs targeting C/EBPα via cMAP

The connectivity map (cMAP, https://clue.io/query) was queried with the common genes that were upregulated in C/EBPα-overexpressing cells and downregulated in *CEBPA* KD cells and C/EBPα-p30-overexpressing cells, and then, the FLT3i (midostaurin, gilteritinib, quizartinib and crenolanib) resistance-associated gene sets were upregulated by C/EBPα (Supplementary Data 6). By choosing a “reverse mode” configuration, we searched for small molecule signatures that could reverse the input signature. Our final query result was displayed as the tau (τ) score (connectivity score) based on the weighted Kolmogorov‒Smirnov statistic. We obtained 5 lists of drugs in the range of −100 to 100 that were pooled together by calculating a “connectivity score”, with reversing (low score) or mimicking (high score) expression signatures with input genes. Finally, the summary drug scores were recalculated with the median statistical analysis. We found that multiple adrenergic receptor-related drugs (including guanfacine) scored below −90. Generally, we considered that τ of +90 or higher and −90 or lower was a strong score, which could be used as a hypothesis for further research.

### CMAP connectivity analysis

The cMAP is a resource for discovering the relationship between diseases, genes and treatments by using disturbances in cellular responses. The cMAP database contains more than one million gene expression signatures (called perturbation signatures) from various cell types treated with small molecule compounds, gene overexpression, and gene knockout reagents. In real experiments, if it was found that diseases or other biological reactions cause changes in gene expression, these changed genes could be compared with the similarity of all perturbation signatures in the database. Perturbation signatures that cause highly similar expression characteristics are called “connections.” Their similar transcriptional effects indicate that they confer similar physiological effects.

The algorithm of cMAP was more complicated (refer to URL for method principle: https://clue.io/connectopedia/cmap_algorithms). In short, the basic unit of cMAP analysis was query (q), which consisted of a set of corresponding genes (generally genes that change under experimental conditions). Each gene in the query had a symbol indicating whether it was upregulated or downregulated. Therefore, each query produces a pair of mutually exclusive genes listed (qup, qdown). The query (q) was compared with each signature in the cMAP reference database (Touchstone) to form a similarity metric score to evaluate connectivity. A high similarity score meant that the signature would cause similar changes in genes, and a low similarity score meant that the signature would cause opposite changes in genes. The result of the query was an ordered list sorted according to the cMAP signature connection score.

The weighted connectivity score (WTCS) uses the weighted Kolmogorov‒Smirnov enrichment score (ES) nonparametric similarity measure (very similar to the GSEA algorithm). WTCS is a two-way combination of ES publicity. Based on the query gene set (qup, qdown) and the reference signature *r*, the calculation formula of the WTCS is as follows:

$${\omega }_{q,r}=\left\{\begin{array}{ll}({{{{{{\rm{ES}}}}}}}_{{{{{{\rm{up}}}}}}}-{{{{{{\rm{ES}}}}}}}_{{{{{{\rm{down}}}}}}})/2 & {{{{{\rm{if}}}}}}\,{{{{{\rm{sgn}}}}}}({{{{{{\rm{ES}}}}}}}_{{{{{{\rm{up}}}}}}}) \, \ne \, {{{{\mathrm{sgn}}}}}({{{{{{\rm{ES}}}}}}}_{{{{{{\rm{down}}}}}}}),\\ 0 \hfill & {{{{{\rm{otherwise}}}}}}\hfill\end{array}\right.$$

ES_up_ is the enrichment of qup in *r*, and ES_down_ is the enrichment of qdown in *r*. WTCS is between −1 and 1. For positively related signatures, it will be positive; for anticorrelated signatures, it will be negative; for irrelevant signatures, it will be close to zero.

To allow the calculated scores to be used universally across cell types, we normalized the scores of these cell lines to account for the effect of differences between these different variables on the overall calculated total score. After we obtained the WTCS values in different cell lines, we normalized each WTCS value to the final normalized connection score (NCS).

Since the final normalized connection score (NCS) of each given gene set for the matching degree of a single drug is an absolute value, we need to obtain a relative ranking score Tau (*τ*) by comparing the NCS score of a single drug with the NCS scores of other drugs in the reference database.

Therefore, our final query result showed the Tau(*τ*) score, and the standardized Tau value measurement range was −100 to 100; if the τ score was 95, it was considered that only 5% of other drugs had an effect on the transcriptome, which was more in line with the genetic changes we input than the current compound.

Generally, if *τ* is +90 or higher or if it is −90 or lower, it was a strong score, which could be used as a hypothesis for further research. Positive scores showed that the corresponding drug could upregulate the upregulated genes that we input. Negative scores showed that the corresponding drugs could downregulate the upregulated genes we input.

To find drugs that inhibit *CEBPA*-related and FLT3i resistance-related genes, we input the common genes that were upregulated in C/EBPα-overexpressing cells and downregulated in *CEBPA* KD cells and C/EBPα-p30-overexpressing cells and upregulated genes of 4 FLTi-resistant primary samples from Vizome into the cMAP database (https://clue.io/). We found that multiple drugs targeting adrenergic receptors (including Guanfacine) scored below −90, so the results suggested that these drugs can downregulate highly expressed genes related to FLT3 inhibitor resistance and *CEBPA* better than 90% of known disturbers (compounds and gene editing) in the database. Therefore, it was predicted that these drugs could have a combined effect with FLT3 inhibitors.

### Statistics and reproducibility

The numbers of animals, cells, and experimental replicates can be found in the figure legends. No statistical method was used to predetermine sample size, and no data were excluded from the analyzes. In cell experiments, the experimenter randomly took different replicates of the same cells and assigned them to control orexperimental groups. In the animal experiment, mice were randomly assigned to the vehicle or administration groups. The investigators were not blinded to allocation during experiments and outcome assessment, but the designer did not inform the experimenter about the drug effects and hypothetical expectations, so the experimenter did not know the biological meaning of the specific grouping. Comparisons of two groups were performed using Student’s *t* test (unpaired, two-tailed) or the Wilcoxon rank sum test. Significance was set at *P* < 0.05. Unless otherwise specified, the results are depicted as the mean ± SEM. Spearman and Pearson tests were performed to assess data distributions. For Kaplan–Meier analysis, the Mantel–Cox test was used. Data were analyzed and plotted using GraphPad Prism 6 software.

## Supplementary information

Supplementary Information

Supplementary Data 1

Supplementary Data 2

Supplementary Data 3

Supplementary Data 4

Supplementary Data 5

Supplementary Data 6

Supplementary Data 7

Supplementary Data 8

Supplementary Data 9

Supplementary Data 10

Supplementary Data 11

## Data Availability

The clinical annotations, whole exome sequencing (WES), RNA-seq data and FLT3i in vitro sensitivity data of the Beat AML cohort were obtained from http://www.vizome.org/. The raw data generated in this study are provided in a Source data file and a supplementary data file. All raw and processed sequencing data generated in this study have been deposited in the NGDC-GSA-human database. The raw sequence data of PDCs are deposited under accession code HRA003991 (https://ngdc.cncb.ac.cn/gsa-human/browse/HRA003991). Cell line sequencing data is deposited under accession code HRA004035 (https://ngdc.cncb.ac.cn/gsa-human/browse/HRA004035). The remaining data are available within the article, Supplementary Information or Source Data file. Source data are provided with this paper.

## Source data

Source Data

## Code availability

Computer codes used for the pathway enrichment circle diagram and bubble chart are available following https://github.com/luoguanghao/FLT3_inhibitor_resistent_project/tree/master.
