# Supplementary material for: Targeting C/EBPα overcomes primary resistance and improves the efficacy of FLT3 inhibitors in acute myeloid leukaemia
Source: Nat Commun. 2023 Apr 5;14:1882. doi: 10.1038/s41467-023-37381-4 (PMC10076519; doi:10.1038/s41467-023-37381-4)
Supplement: Supplementary file 1 — Supplementary Information [file 41467_2023_37381_MOESM1_ESM.pdf]

Supplementary Information

Targeting C/EBPα overcomes primary resistance and improves the efficacy of FLT3 inhibitors in acute myeloid leukaemia

Hanlin Wang[1,2,3], Guanghao Luo[1,3,4], Xiaobei Hu[1,5], Gaoya Xu[1,6], Tao Wang[7], Minmin Liu[1,8], Xiaohui Qiu[1,5], Jianan Li[1,6], Jingfeng Fu[1,3], Bo Feng[1,9], Yutong Tu[1,3], Weijuan Kan[1], Chang Wang[1], Ran Xu[1,6], Yubo Zhou[1,3,5,6*], Jianmin Yang[7*], Jia Li[1,2,3,4,5,6,9*]

Affiliations:

[1]State key Laboratory of Drug Research, Shanghai Institute of Materia Medica, Chinese Academy of Sciences; Shanghai, 201203, China.

[2]College of Pharmacy, Fudan University; Shanghai, 210023, China.

[3]University of Chinese Academy of Sciences; Beijing 100049, China.

[4]School of Pharmaceutical Science and Technology, Hangzhou Institute for Advanced Study, University of Chinese Academy of Sciences; Hangzhou, 310000, China.

[5]Zhongshan Institute for Drug Discovery, Shanghai Institute of Materia Medica, Chinese Academy of Sciences; Guangdong, 528400, China.

[6]School of Chinese Materia Medica, Nanjing University of Chinese Medicine, Nanjing, 210023, China.

[7]Department of Hematology, Changhai Hospital, Naval Medical University, Shanghai, 200433, China.

[8]School of Pharmaceutical Science, Jiangnan University, Wuxi, 214122, China

[9]School of Life Science and Biopharmaceutics, Shenyang Pharmaceutical University, No.103 Wenhua Road, Shenyang, Liaoning, China

[*] **Corresponding Authors:**

Yubo Zhou, State Key Laboratory of Drug Research, Shanghai Institute of Materia Medica, Chinese Academy of Sciences; Shanghai, 201203, China. Phone: 86-21-50801313; E-mail: ybzhou@simm.ac.cn

Jianmin Yang, Department of Hematology, Changhai Hospital, Naval Medical University, Shanghai, 200433, China. Phone: 86-21-31161280; E-mail: chyangjianmin@163.com

Jia Li, State Key Laboratory of Drug Research, Shanghai Institute of Materia Medica, Chinese Academy of Sciences; Shanghai, 201203, China. Phone: 86-21-50806600; E-mail: jli@simm.ac.cn

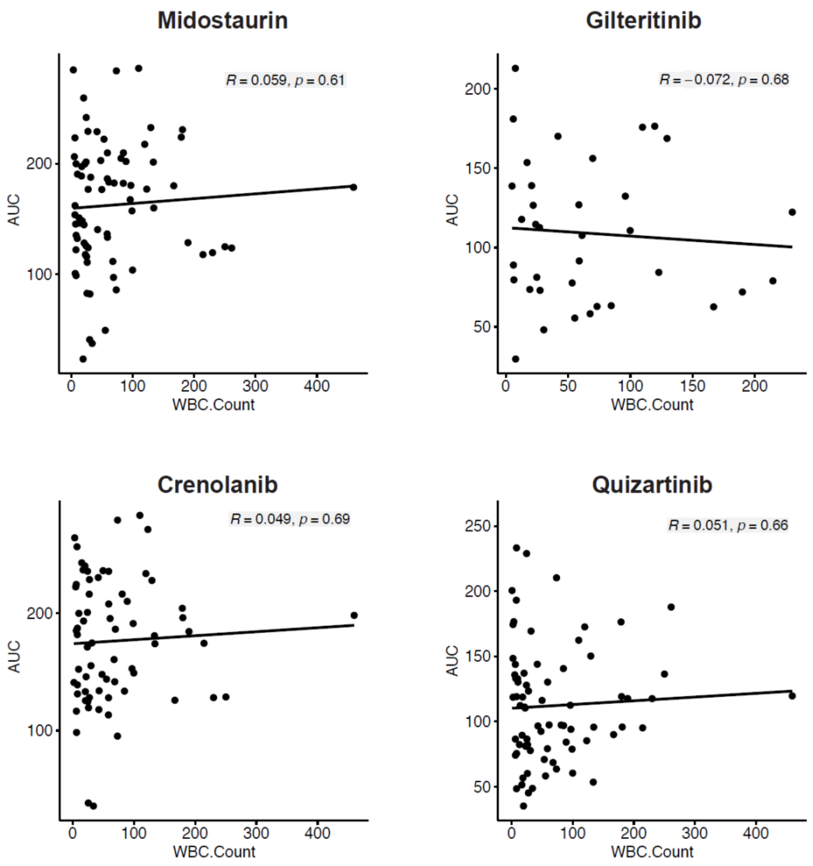

Supplementary Figure 1. The correlation between WBC count and FLT3i sensitivity. The correlation between AUC of midostaurin, gilteritinib, crenolanib and quizartinib against FLT3-ITD AML patients in Vizome and the WBC count is determined by the Spearman tests, respectively.

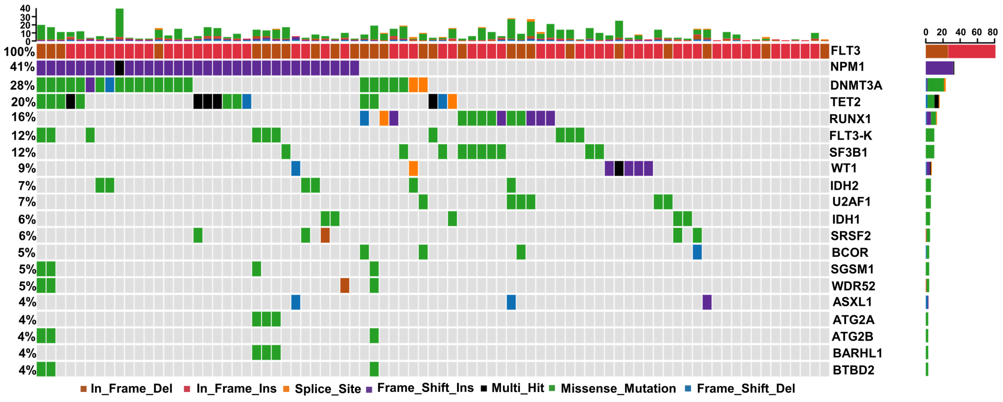

Supplementary Figure 2. Mutational landscape of FLT3-ITD patients in the Vizome database.

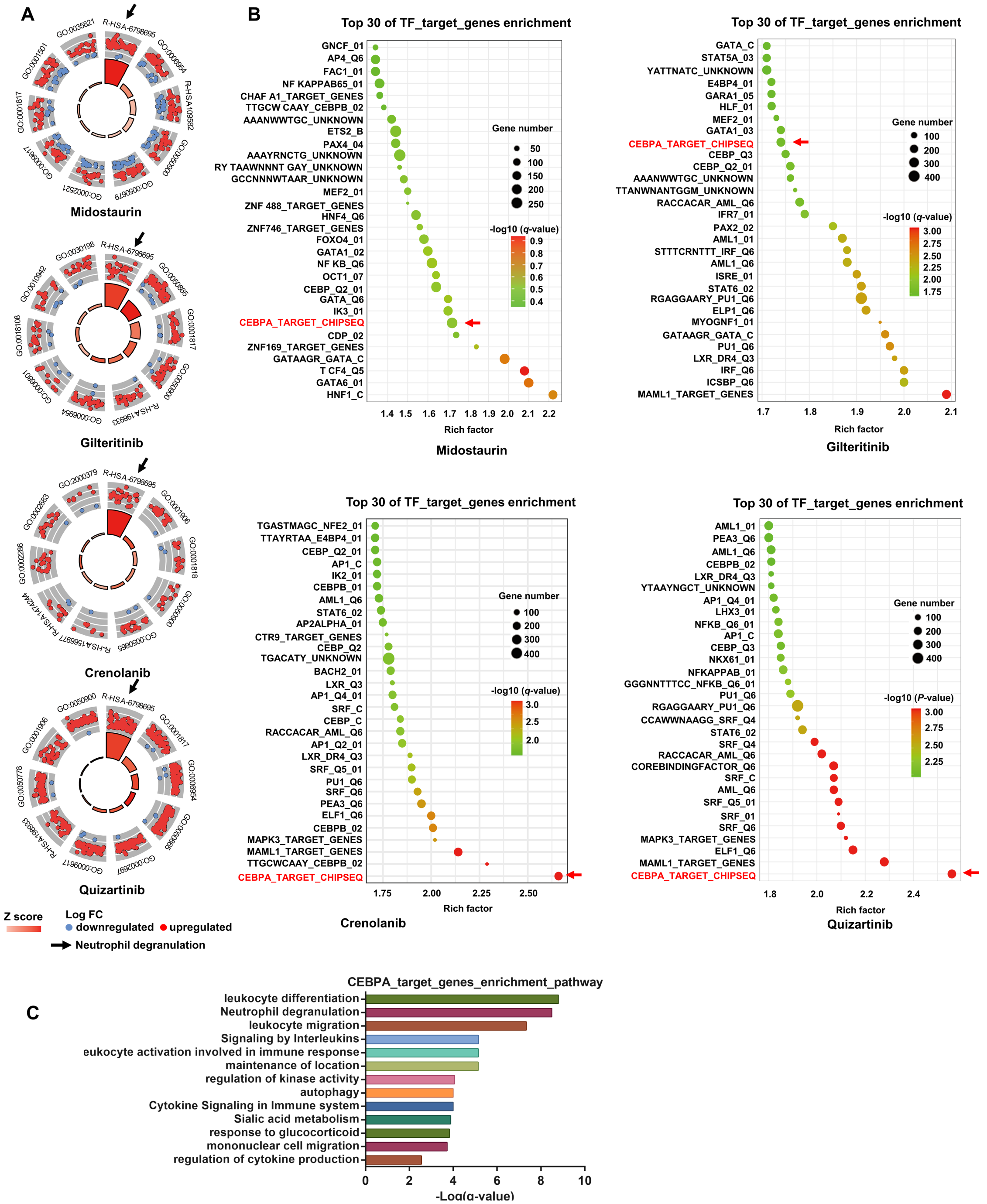

Supplementary Figure 3. Gene expression patterns in FLT3i-resistant patients. (**A**) Functional enrichment analysis of the differential signaling pathways in 4 FLT3i-resistant FLT3-ITD AML patients in the Vizome database. (**B**) Transcription factor enrichment analysis in 4 FLT3i-resistant FLT3-ITD AML patients. (**C**) Enrichment analysis of C/EBPα-targeting gene pathways by Metascape.

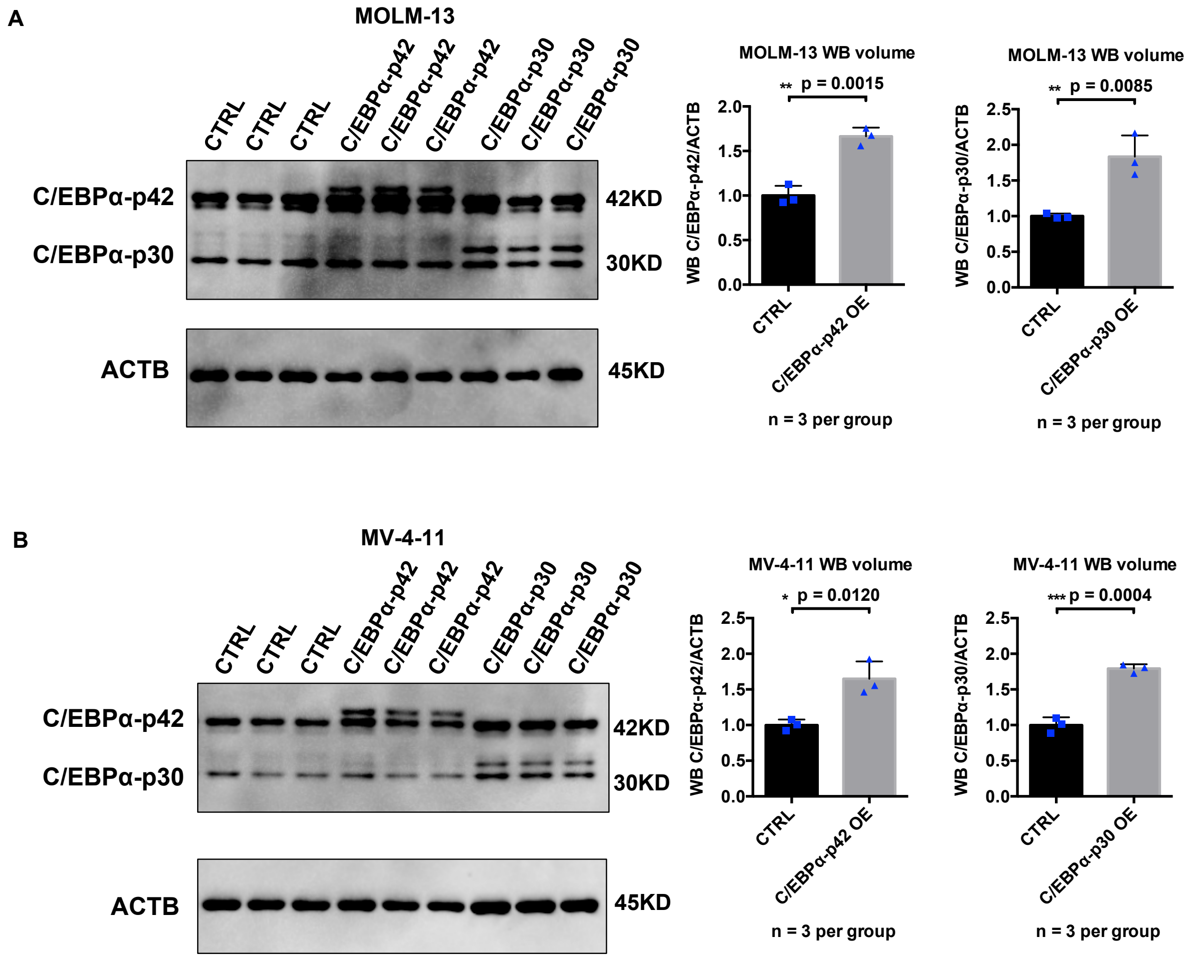

Supplementary Figure 4. WB quantification of C/EBPα-p42 and C/EBPα-p30 overexpression in MOLM-13 and MV-4-11. (**A**). WB quantification of C/EBPα-p42 and C/EBPα-p30 overexpression in MOLM-13 in three independent experiments. Relative protein level (C/EBPα-p42/ACTB) in per group are Means ± SEM. Significance was analyzed by equal variance two-tailed *t* test.*$P < 0.05$, **$P < 0.01$, *** $P < 0.001$. (**B**) WB quantification of C/EBPα-p42 and C/EBPα-p30 overexpression in MV-4-11 using three independent experiments. Relative protein level (C/EBPα-p42/ACTB) in per group are Means ± SEM. Significance was analyzed by equal variance two-tailed *t* test.*$P < 0.05$, **$P < 0.01$, *** $P < 0.001$.

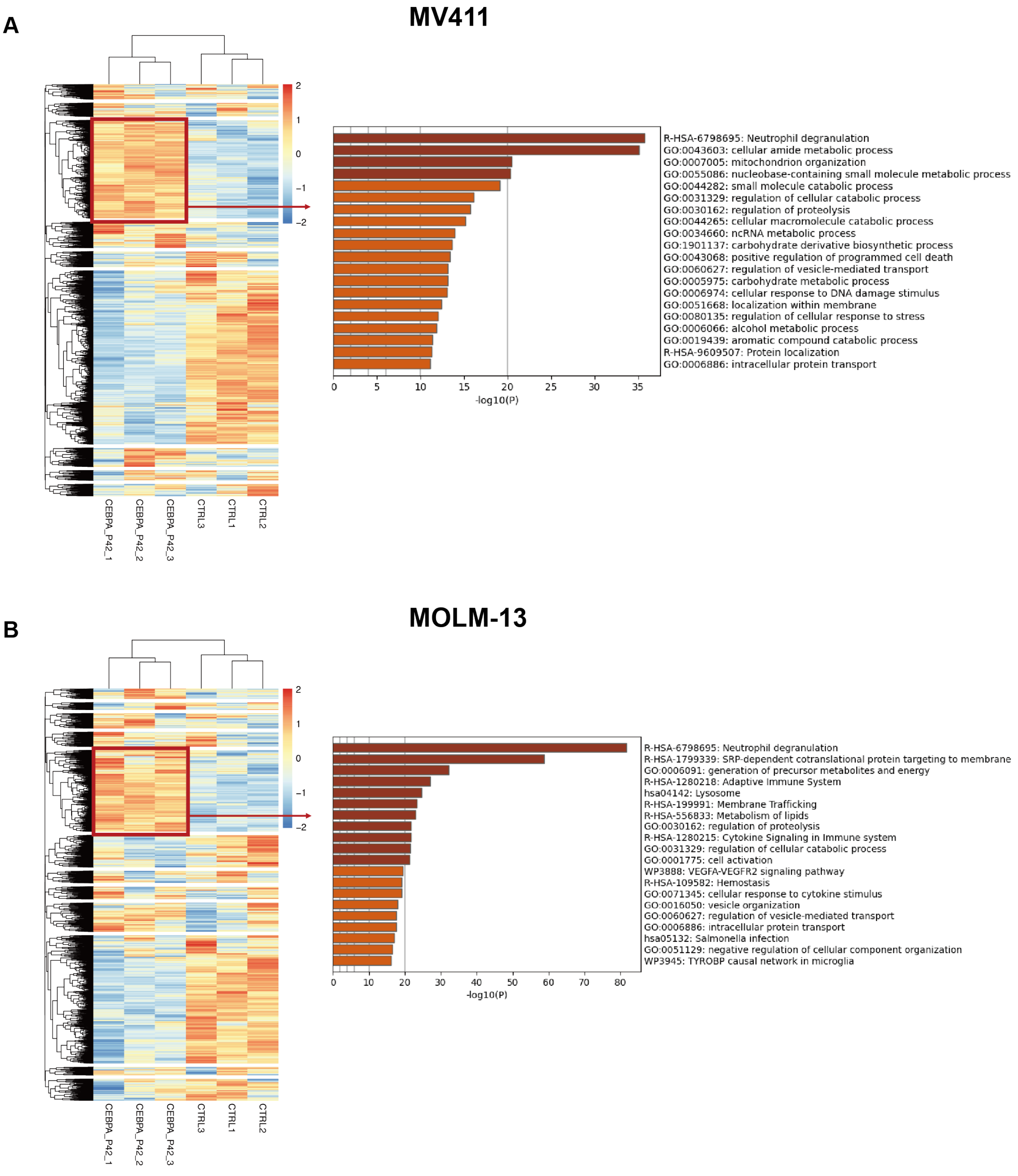

Supplementary Figure 5. Unsupervised heatmap of gene expression profiles for C/EBPα-p42 overexpression in MV-4-11 and MOLM-13 cells. In (**A**) MV-4-11 and (**B**) MOLM-13, unsupervised heatmaps of gene expression profiles for C/EBP α-p42 overexpression and CTRL samples, and the up-regulated genes are functional enriched. Significance was determined by Fisher's exact test, and p-value was adjusted for multiple comparisons.

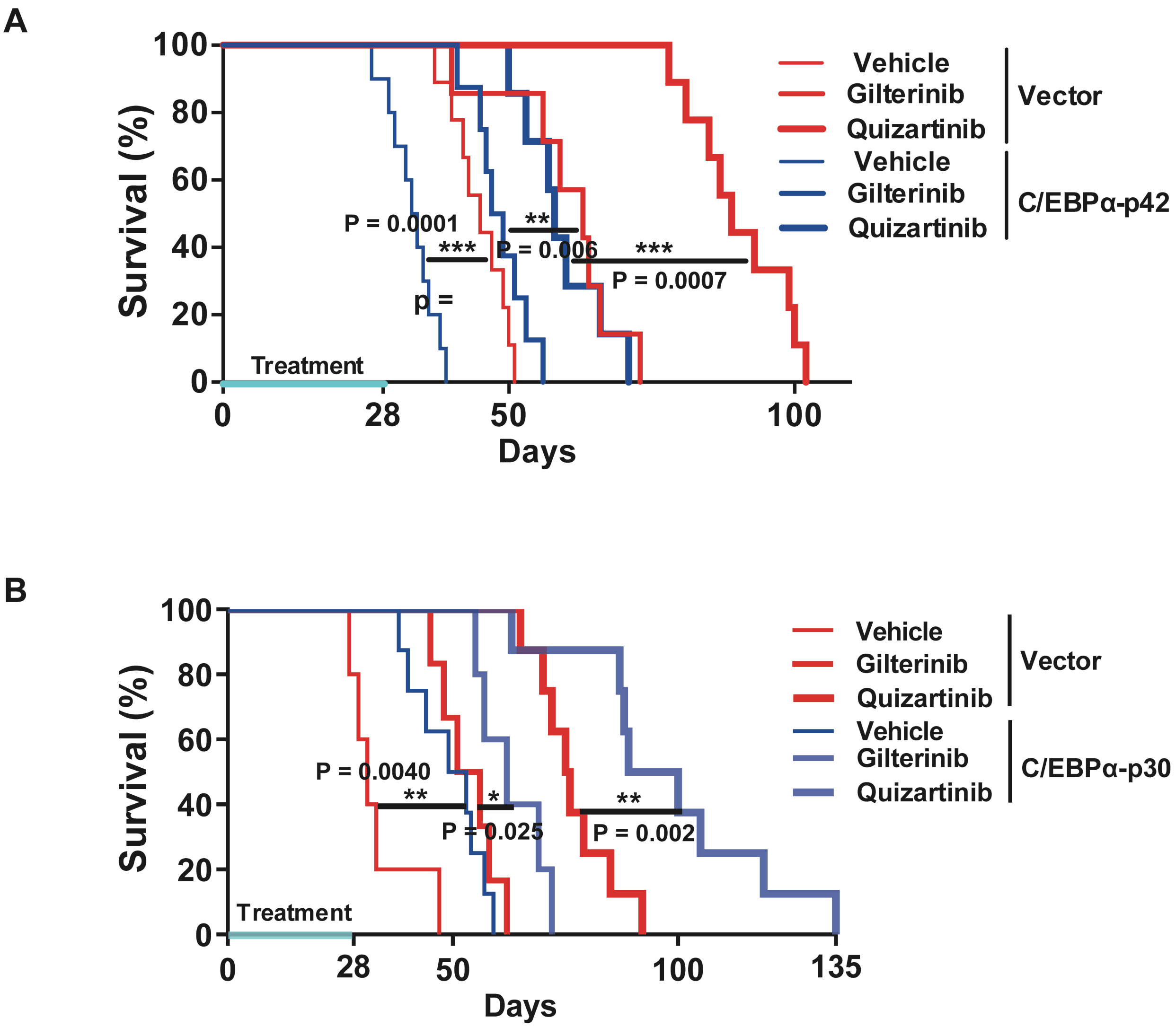

Supplementary Figure 6. The effect of FLT3i in the MV-4-11 in vivo disseminated xenograft model with C/EBPα-p42 or p30 overexpression. (**A**) Survival curve of B-NDG mice xenografted with MV-4-11 cells with or without C/EBPα-p42 overexpression and treated with gilteritinib (3 mg/kg, n=10 mice), quizartinib (3 mg/kg, n=10 mice) or vehicle control (22% Beta-CD, n = 20 mice). Significance was analyzed by *logrank* test. *$P < 0.05$, **$P < 0.01$, *** $P < 0.001$. (**B**) Survival curve of B-NDG mice xenografted with MV-4-11 with or without C/EBPα-p30 overexpression and treated with gilteritinib (3 mg/kg, n=10 mice), quizartinib (3 mg/kg, n=10 mice), or vehicle control (22% Beta-CD, n = 20 mice). Significance was analyzed by *logrank* test. *$P < 0.05$, **$P < 0.01$, *** $P < 0.001$.

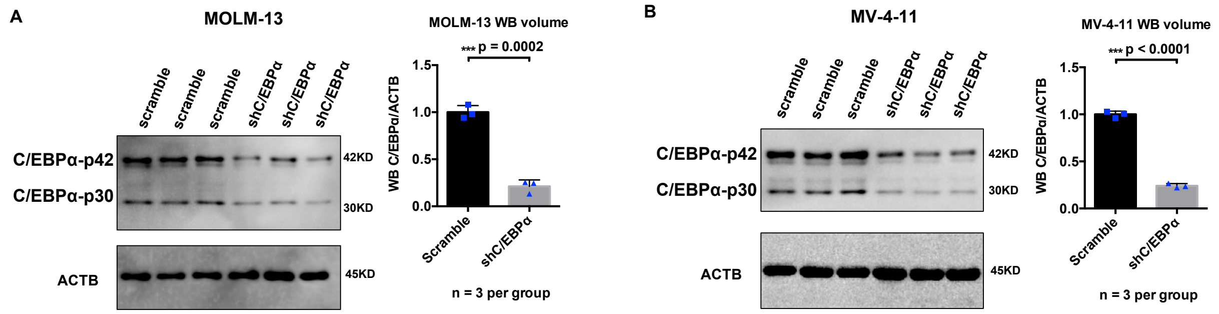

Supplementary Figure 7. WB quantification of *CEBPA* knockdown in MOLM-13 and MV-4-11. (**A**) WB quantification of *CEBPA* knockdown in MOLM-13 with three independent experiments. Relative protein level (C/EBPα/ACTB) in per group are Means ± SEM. Significance was analyzed by equal variance two-tailed *t* test.*$P < 0.05$, **$P < 0.01$, *** $P < 0.001$. (**B**) WB quantification of *CEBPA* knockdown in MV-4-11 with three independent experiments. Relative protein level (C/EBPα/ACTB) in per group are Means ± SEM. Significance was analyzed by equal variance two-tailed *t* test.*$P < 0.05$, **$P < 0.01$, *** $P < 0.001$.

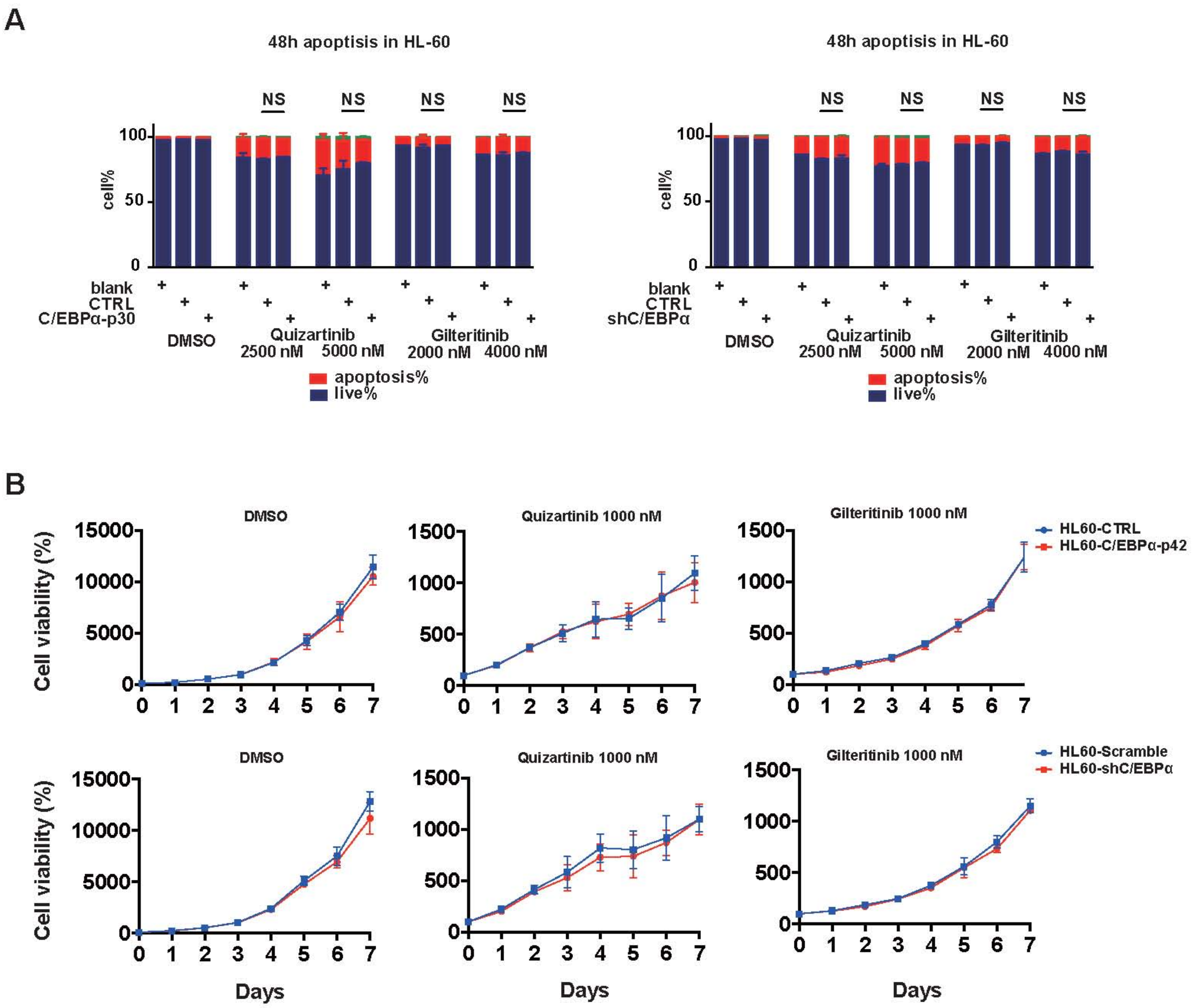

Supplementary Figure 8. The effect of FLT3i alone in HL-60 cells overexpressing C/EBPα-p42 and sh*CEBPA*. (**A**) Apoptosis statistics of the treatment of FLT3i in HL60 for 48 hr. n= 3 independent experiments. Data are Means ± SEM. Significance was analyzed by equal variance two-tailed *t* test.**P* < 0.05, ***P* < 0.01, *** *P* < 0.001. (**B**) Growth curve of HL60 in the treatment of FLT3i for 7 days. n = 3 independent experiments. Data are Means ± SEM. Significance was analyzed by equal variance two-tailed *t* test.**P* < 0.05, ***P* < 0.01, *** *P* < 0.001.

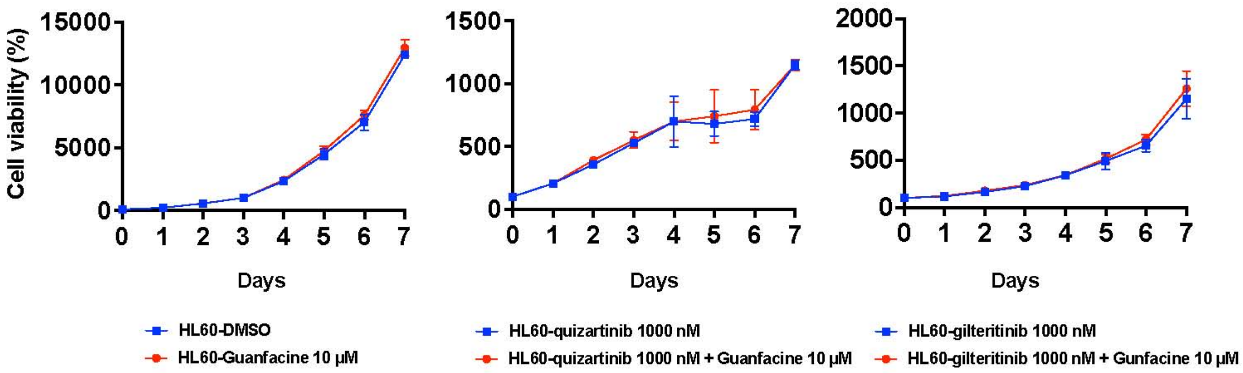

Supplementary Figure 9. The effect of Guanfacine alone or in combination with FLT3i in HL-60 cell growth. Growth curve of HL60 in the treatment of guanfacine alone and combination with FLT3i. n = 3 independent experiments. Data are Means ± SEM. Significance was analyzed by equal variance two-tailed *t* test.**P* < 0.05, ***P* < 0.01, *** *P* < 0.001.

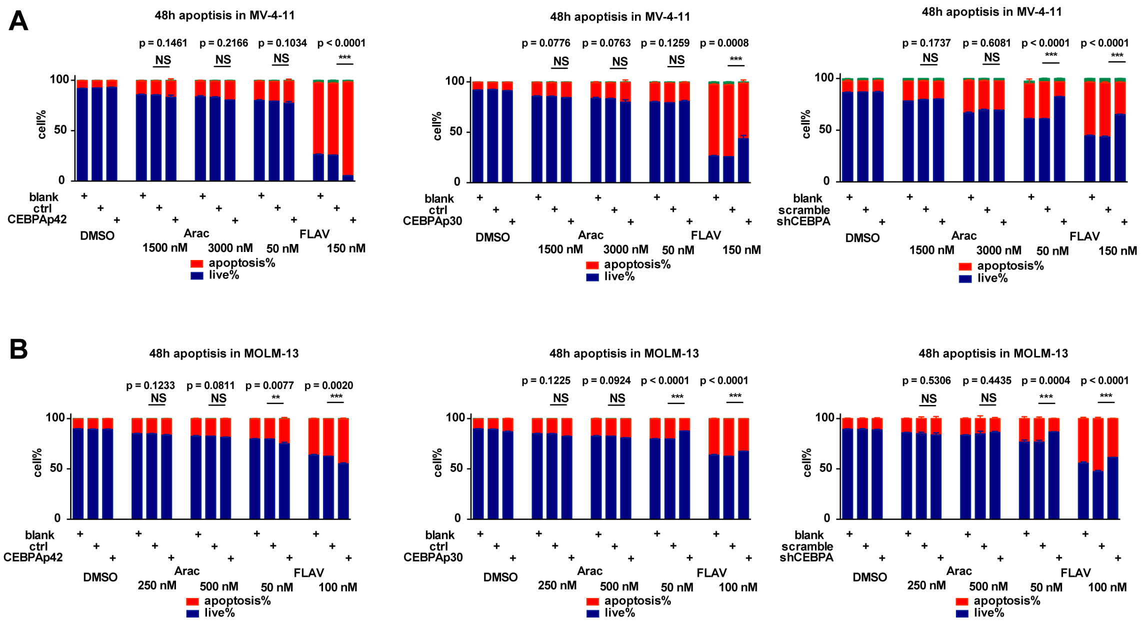

Supplementary Figure 10. The apoptosis induction of cytarabine and flavopiridol on MOLM-13 and MV-4-11 with C/EBPα-p42, p30 and sh*CEBPA* overexpression. Apoptosis statistics of cytarabine, flavopiridol treatment for 48hr with the indicated concentration in MV-4-11 (A) and MOLM-13 (B). n= 3 independent experiments. Data are Means ± SEM. Significance was analyzed by equal variance two-tailed *t* test.**P* < 0.05, ***P* < 0.01, *** *P* < 0.001.

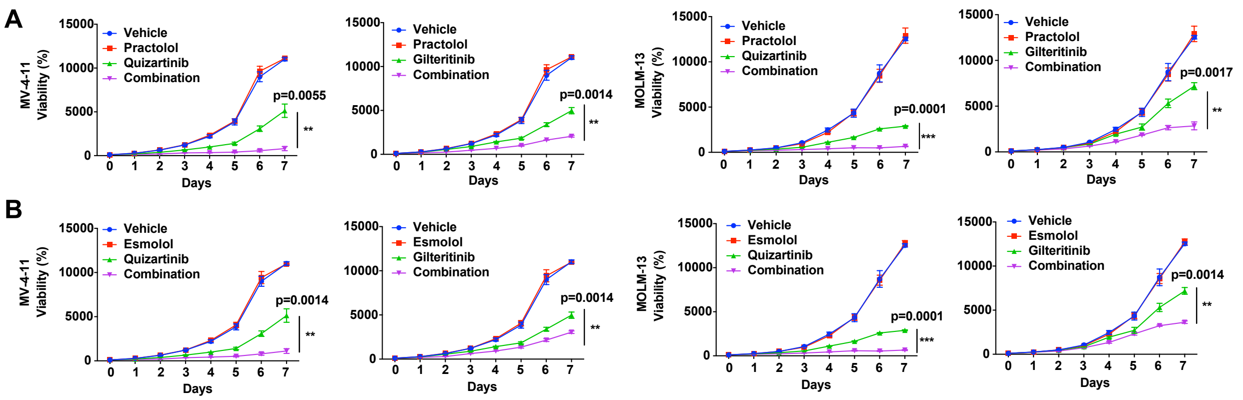

Supplementary Figure 11. The combination of drugs targeting adrenergic receptors with FLT3i in FLT3-ITD cells. The cell growth curve of MV-4-11 and MOLM-13 cells treated with quizartinib or gilteritinib in the absence or presence of (**A**) practolol and (**B**) esmolol. n = 3 independent experiments. Data are Means ± SEM. Significance was analyzed by equal variance two-tailed *t* test.**P* < 0.05, ***P* < 0.01, *** *P* < 0.001.

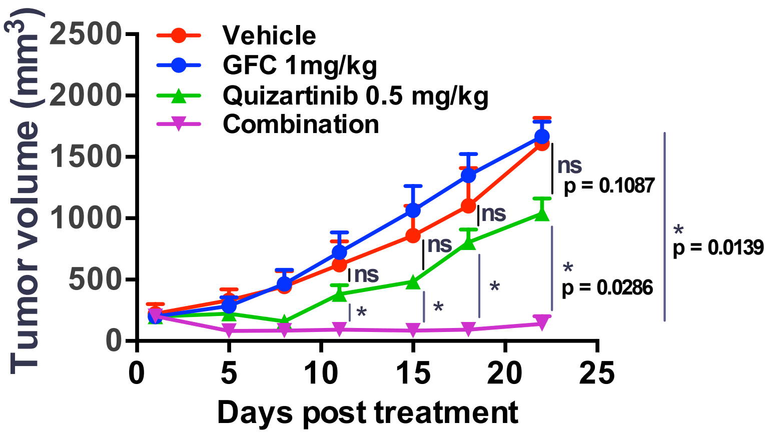

Supplementary Figure 12. The effect of FLT3i combined with Guanfacine in the MV-4-11 in vivo subcutaneous model. The tumor growth of MV-4-11 cells treated with vehicle (22% Beta-CD, n=10 mice), quizartinib (0.5 mg/kg, n=5 mice), GFC (1 mg/kg, n=5 mice), or the combination (n=5 mice) in the subcutaneous transplantation tumor model. Data are Means ± SEM. Significance was analyzed by Wilcox test.**P* < 0.05, ***P* < 0.01, *** *P* < 0.001.

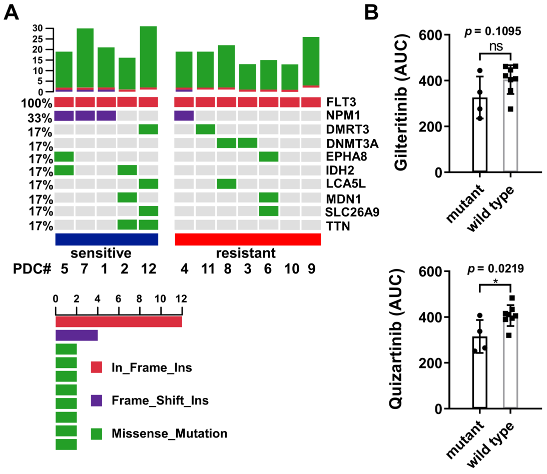

Supplementary Figure 13. Mutation and transcriptional patterns of 12 FLT3-ITD patients and response to FLT3i. (A) Mutation patterns of 12 FLT3-ITD patients. (B)The AUC of quizartinib and gilteritinib in NPM-1 wild-type and mutant FLT3-ITD patient cells. n=4 patient samples in mutant group, n=8 patient samples in wild type group. Data are Means ± SEM. Significance was analyzed by equal variance two-tailed t test.*P < 0.05, **P < 0.01, *** P < 0.001.

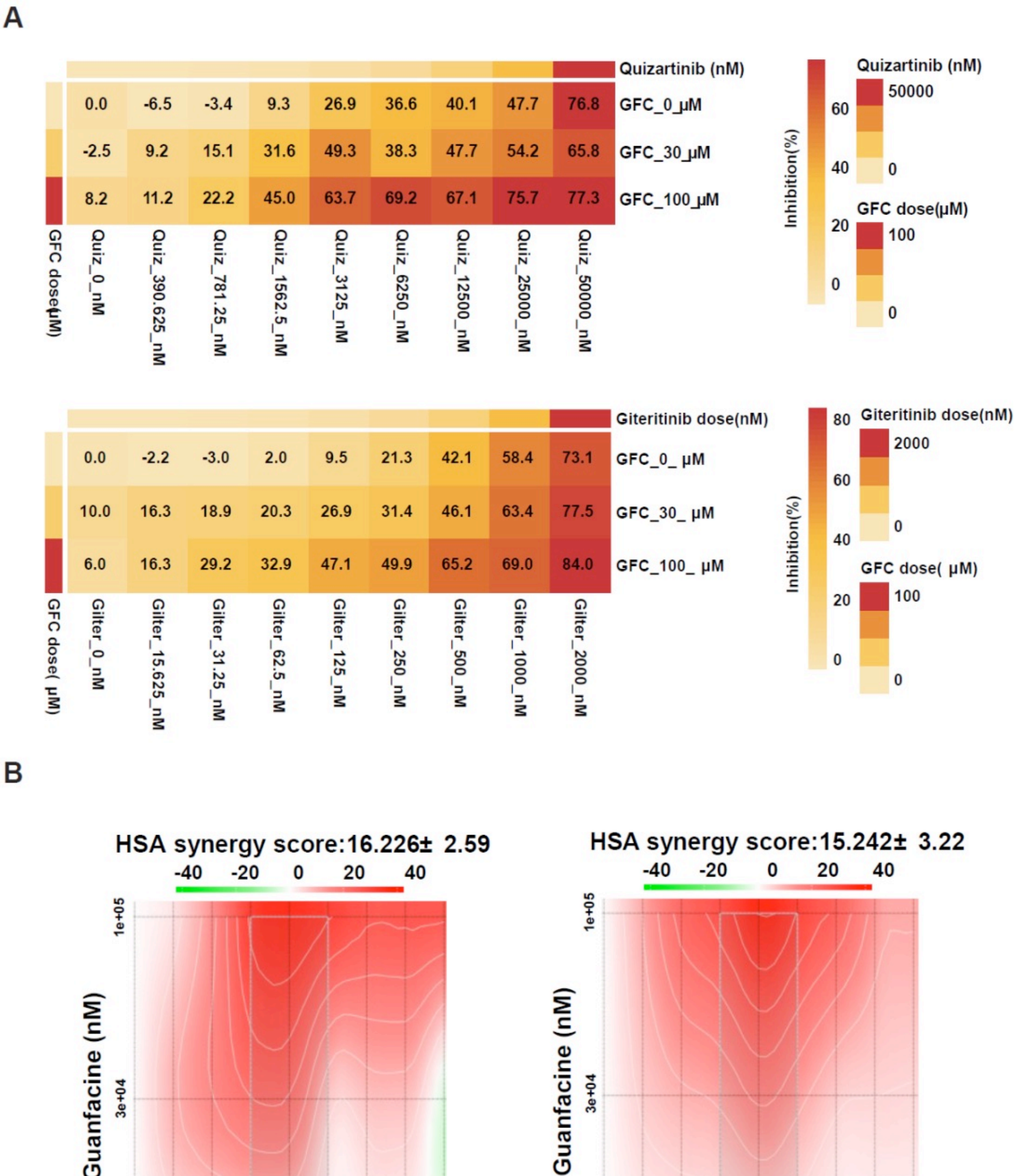

Supplementary Figure 14. The synergy score of the combination of GFC and FLT3i in PDC. (A) The inhibition rate of GFC and two FLT3 inhibitors (Quizartinib and Gilteritinib) alone and in combination in PDC3#. (B)The HSA algorithm was used to calculate the synergy score between GFC and FLT3i in PDC3#.

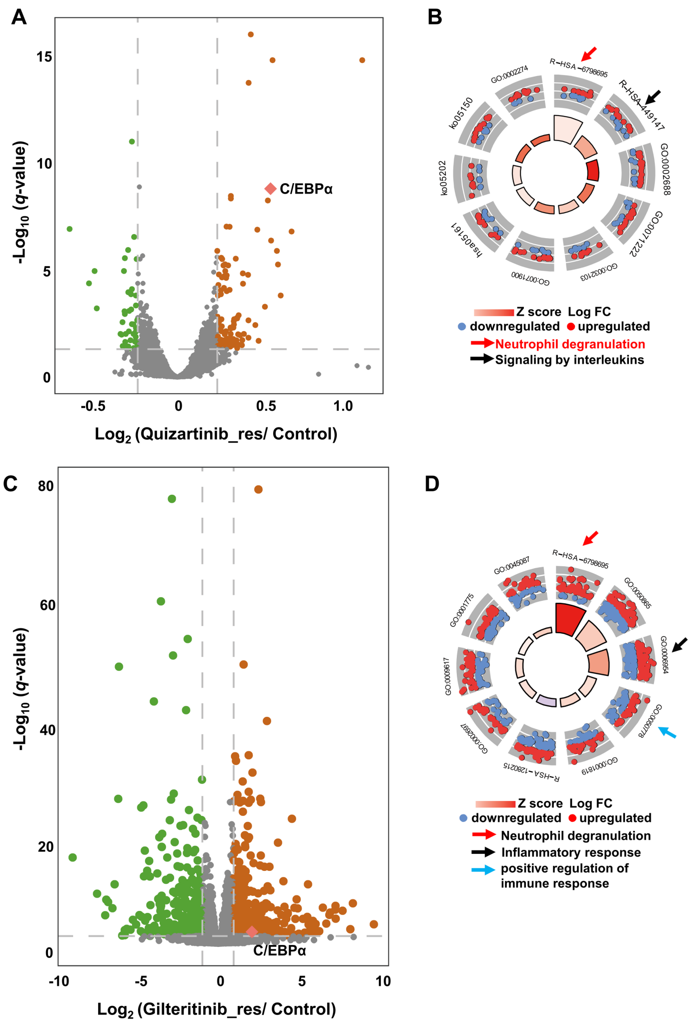

Supplementary Figure 15. FLT3i treatment induces C/EBPα expression and activation. The engineered primary CD34^{+} human cell lines expressing MLL-AF9 and FLT3-ITD were treated with 0.3 nM quizartinib for 12 h. (**A**) The differential gene expression, and (**B**) regulated biological pathways were analyzed. MOLM14 cells cultured with 10 ng/mL of FGF2 or FL were treated with 100 nM gilteritinib for 48 h. (**C**) The differential gene expression and (**D**) regulated biological pathways were analyzed.

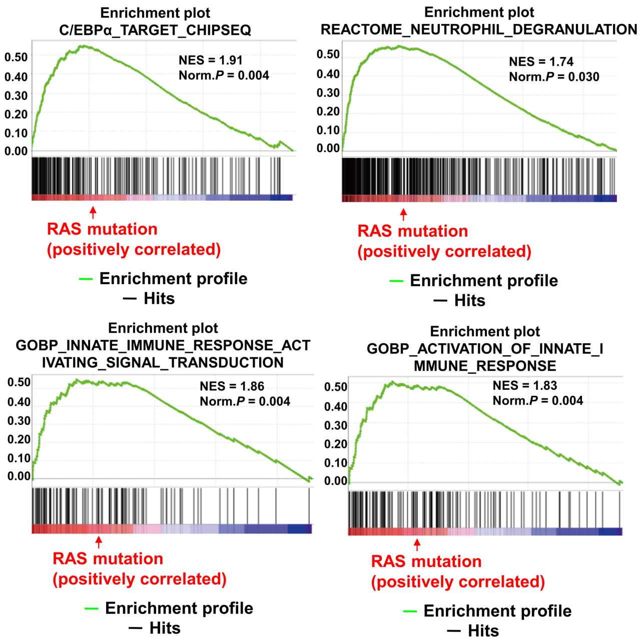

Supplementary Figure 16. GSEA enrichment profiles of C/EBPα target genes and related pathways in RAS mutant AML patients in Vizome. Significance of GSEA results was determined by the two-sided permutation test, and p-value was adjusted for multiple comparisons.

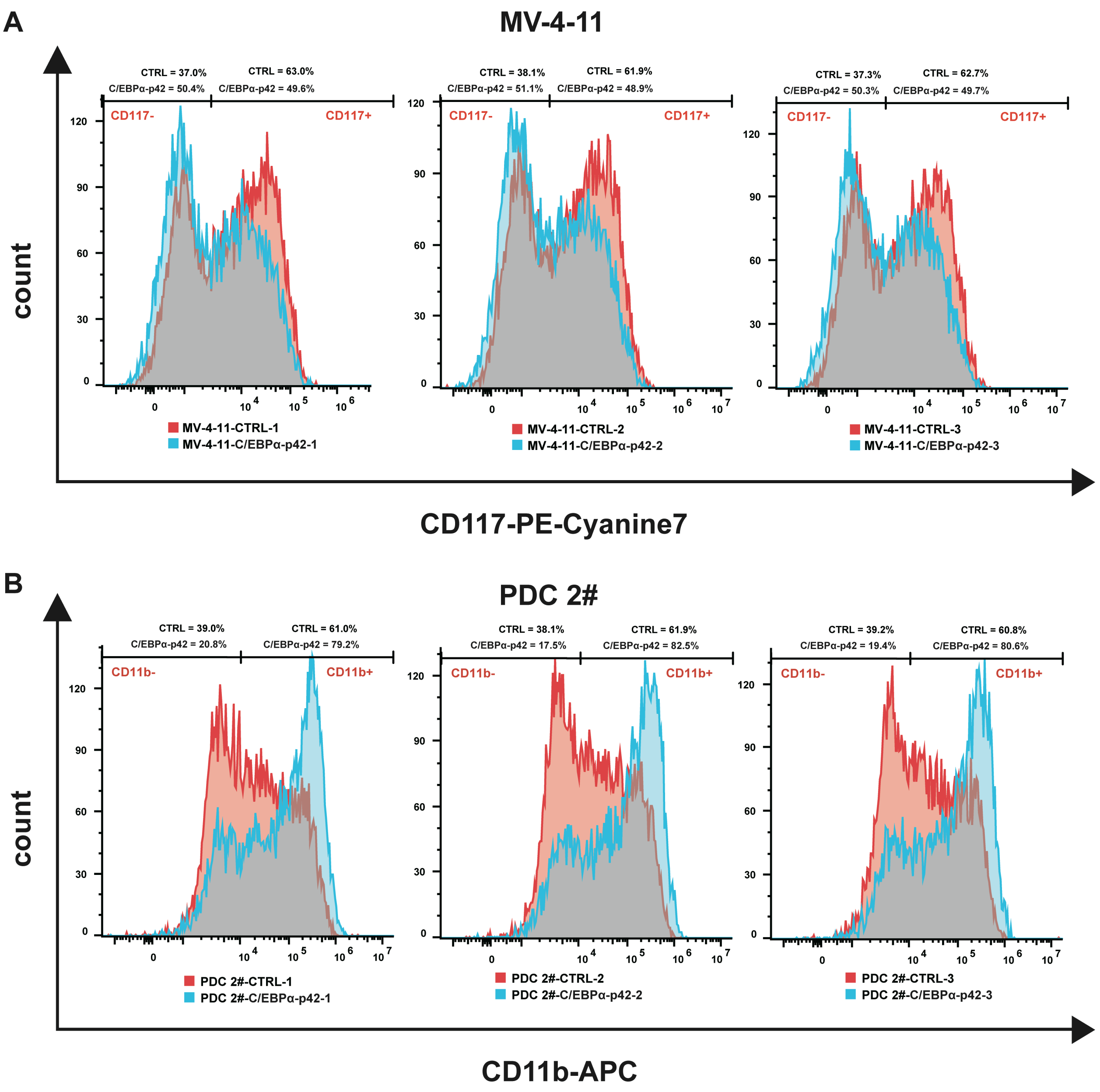

Supplementary Figure 17. The effect of C/EBPα-p42 on cell differentiation in MV-4-11 and PDC 2# with flow cytometry. (A) 500,000 MV-4-11 cells with or without C/EBPα-p42 expression were cultured with CD117 (c-Kit) monoclonal Antibody (2B8, PE-Cyanine7, eBioscience) for 30min, then washed with PBS, then analyzed with flow cytometry (CytoFLEX flow cytometer, Beckman Coulter, Inc) using 488 nm excitation and 780 nm emission filter. (B) 500,000 PDC 2# cells with or without C/EBPα-p42 were cultured with CD11b monoclonal Antibody (ICRF44, APC, eBioscience)) for 30min, then washed with PBS, then analyzed with flow cytometry (CytoFLEX flow cytometer, Beckman Coulter, Inc) using 630nm excitation and 60 nm emission filter. n= 3 independent experiments.

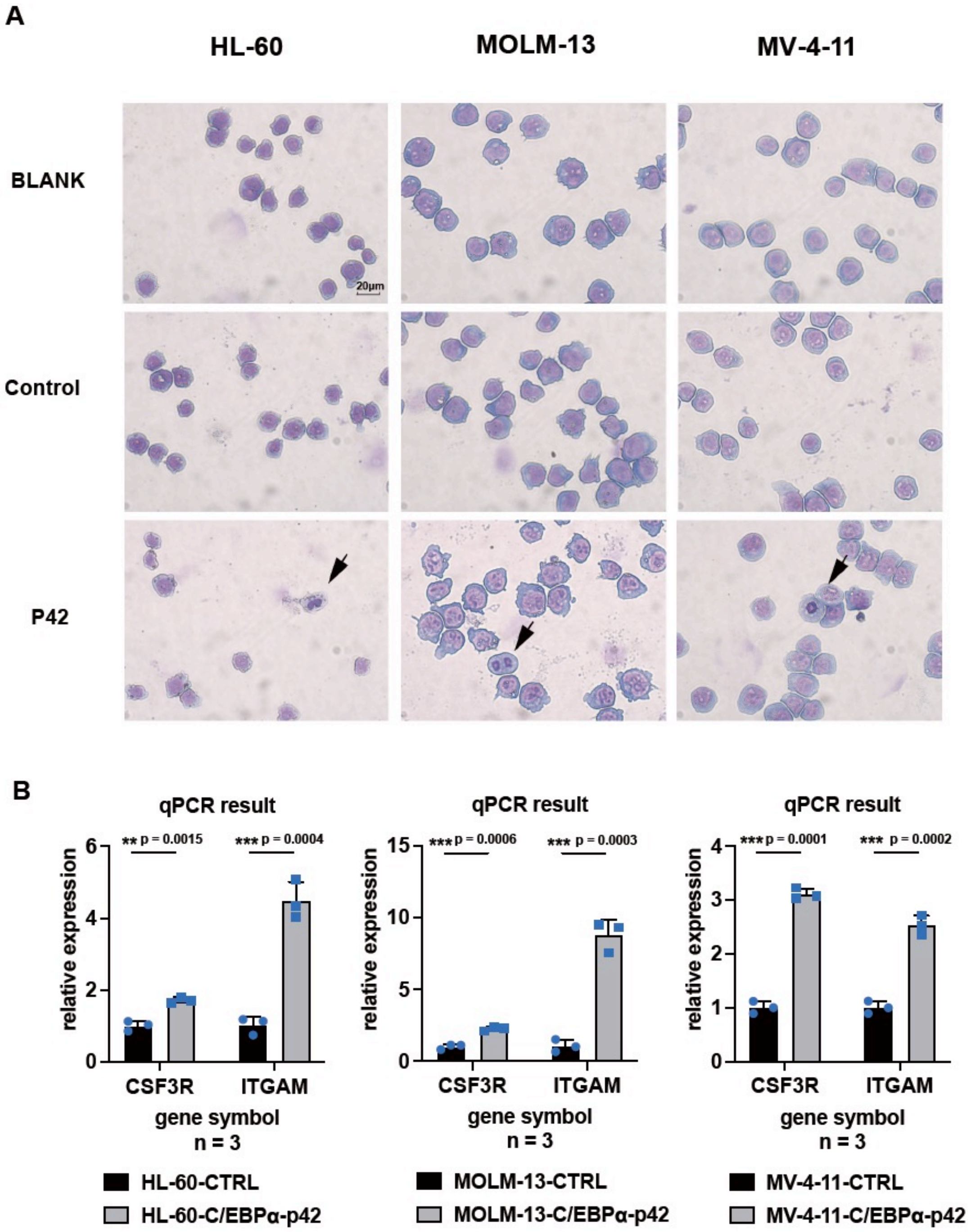

Supplementary Figure 18. The effect of C/EBPα-p42 on cell differentiation of MV-4-11, MOLM13 and HL-60 using Wright-Giemsa staining and Q-PCR. (A) Cells marked with arrows are considered to have a differentiated phenotype. MOLM-13, MV-4-11, HL60 cells with or without C/EBPα-p42 expression were cultured and stained with Fast Wright's-Giemsa Stain Kit, following the protocol in method, photographed in 600x microscope. The experiment was repeated 2 times with similar results. **(B)** qPCR analysis of CSF3R, ITGAM mRNA expression in MOLM-13, MV-4-11, HL60 cells with or without C/EBPα-p42 expression. n= 3 independent

experiments. Data are Means ± SEM. Significance was analyzed by equal variance two-tailed *t* test. *$P < 0.05$, **$P < 0.01$, *** $P < 0.001$.

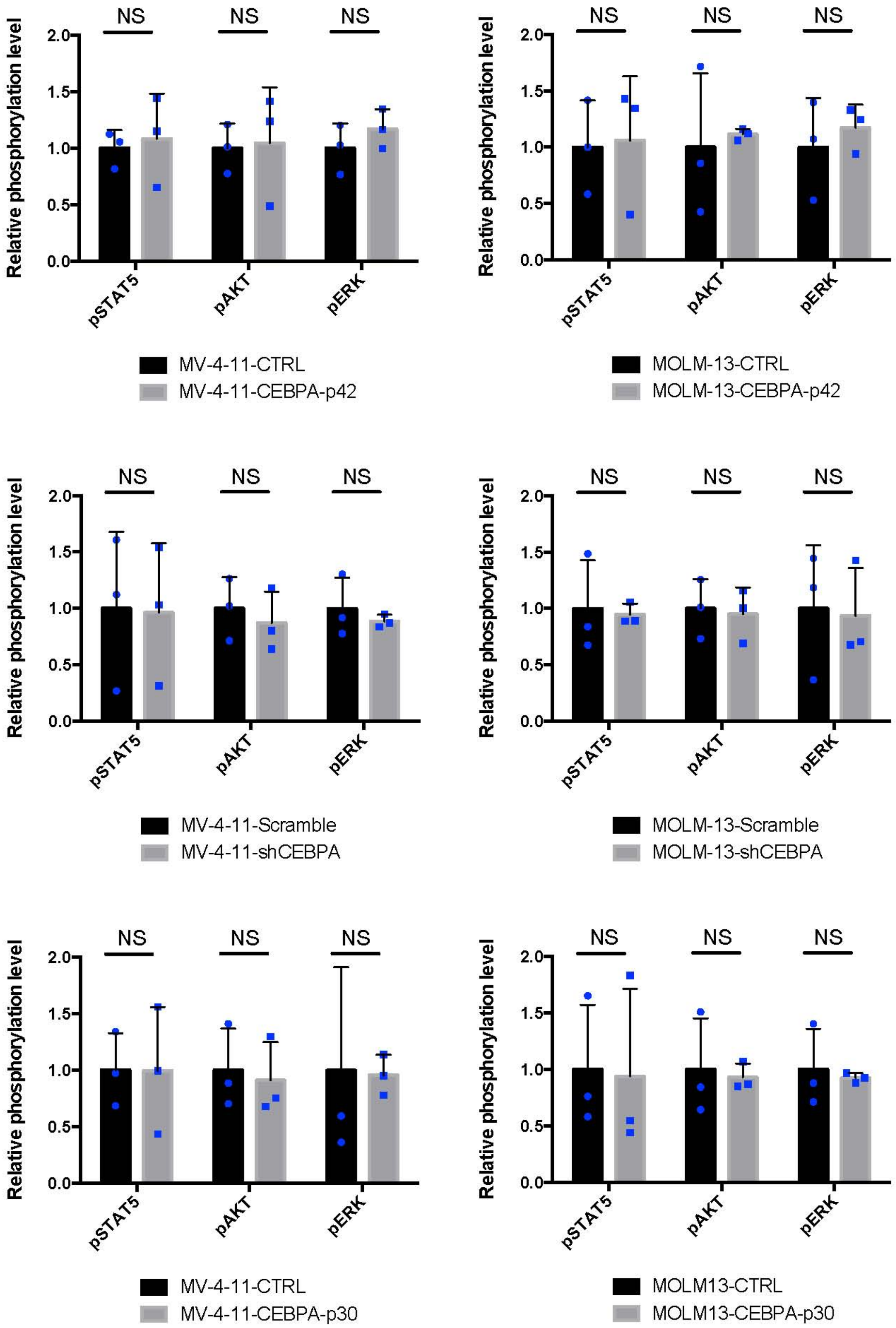

Supplementary Figure 19. WB quantification of C/EBPα-p42, p30, and sh*CEBPA* on the phosphorylation of STAT5, AKT, and ERK in MOLM-13 and MV-4-11. n= 3 independent experiments. Data are Means ± SEM. Significance was analyzed by equal variance two-tailed *t* test.$^*P < 0.05$, $^{**}P < 0.01$, $^{***}P < 0.001$.

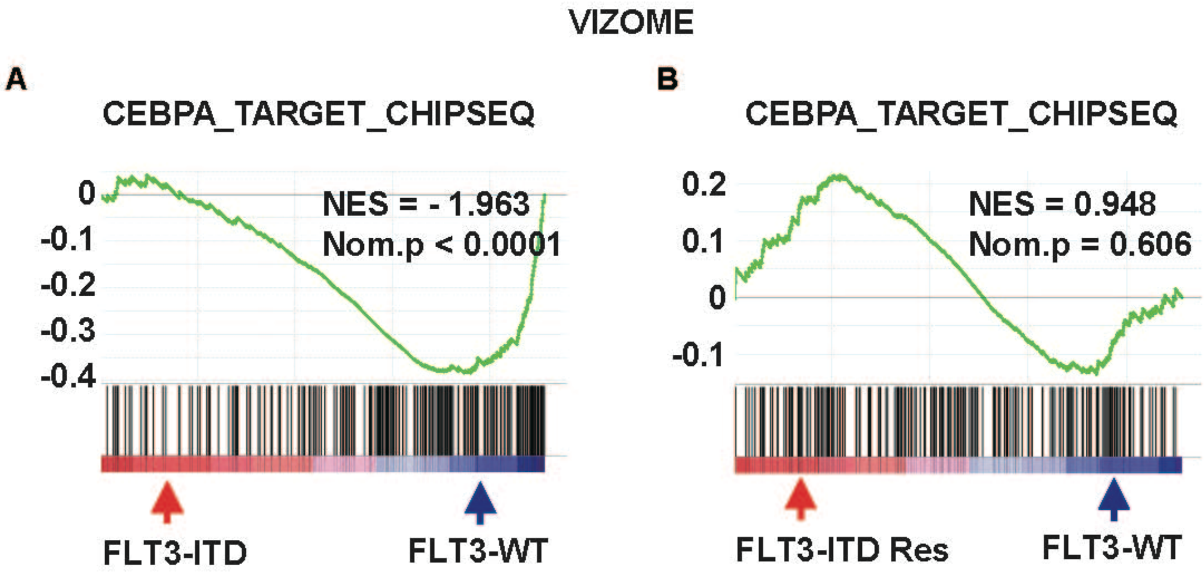

Supplementary Figure 20. The activity of C/EBPα in FLT3-ITD, FLT3i resistant, and FLT3 WT PDC in Vizome. (**A**) GSEA enrichment profiles (included p-value and enrichment score) of C/EBPα target genes of the differential transcriptome of FLT3-ITD samples and FLT3-WT samples in Vizome. (**B)** GSEA enrichment profiles (included p-value and enrichment score) of C/EBPα target genes of the differential transcriptome of FLT3i resistant FLT3-ITD samples and FLT3-WT samples in Vizome. Significance of GSEA results was determined by the two-sided permutation test, and p-value was adjusted for multiple comparisons.

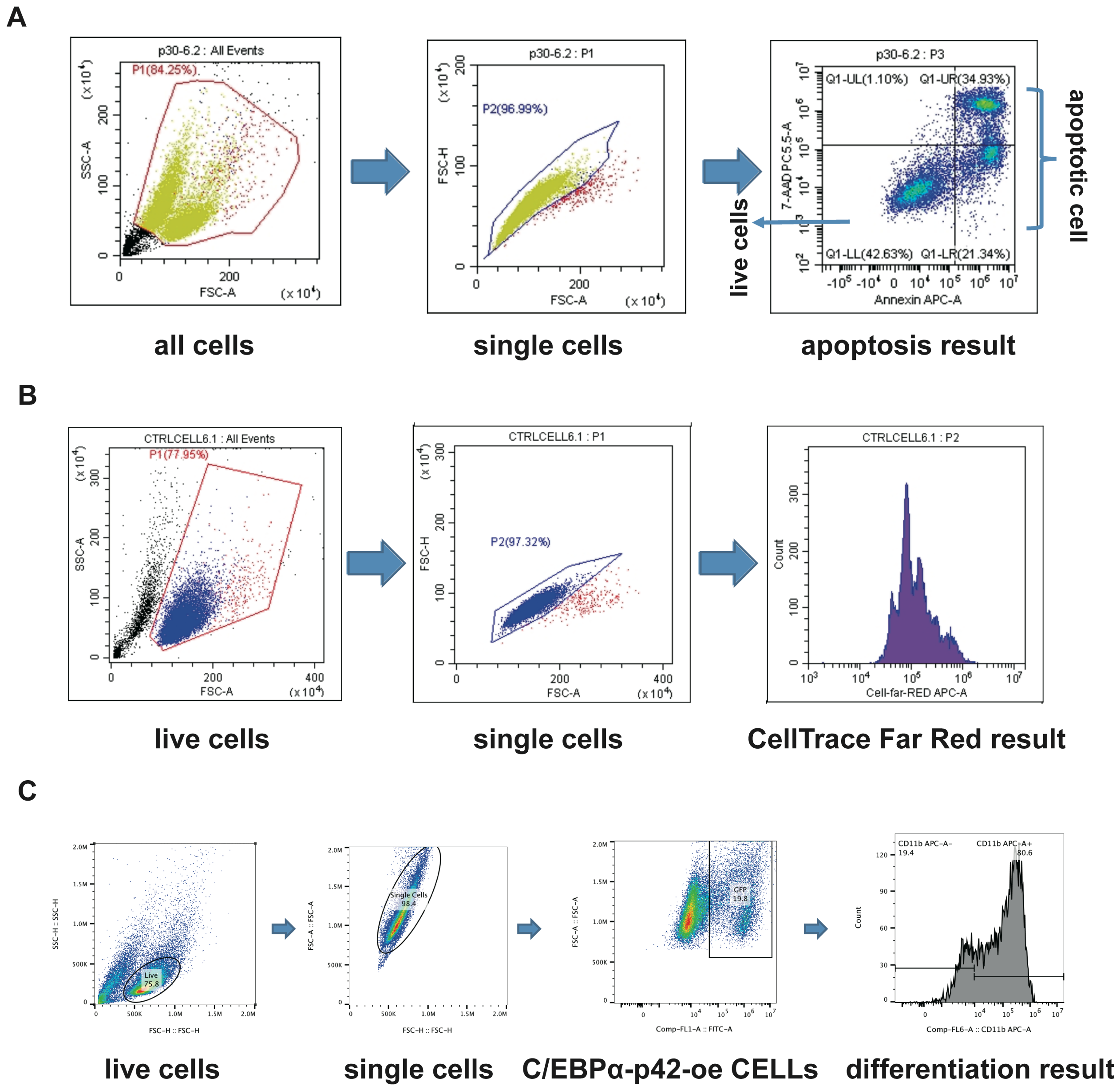

Supplementary Figure 21. FACS sequential gating strategies of (A) apoptosis analysis, (B) CellTrace^{TM} Far Red analysis, (C) cell differentiation analysis.

Supplementary Table 1.
4 drugs targeting adrenergic receptors

Name	Mechanism	Routes of Administration	Development Phase	Indication	Number of approved countries
esmolol	β1 adrenergic receptor antagonist	i.v	approved	hypertension, Arrhythmia, Diabetic foot ulcer	4
practolol	β1 adrenergic receptor antagonist	p.o, i.v	withdrawn	hypertension	0
guanfacine	α2 adrenergic receptor antagonist	p.o	approved	ADHD, hypertension	17
ST-91	α2 adrenergic receptor antagonist	s.c	preclinical	hypertension	0

Supplementary Table 2.
The IC50 of quizartinib and gilteritinib in PDC with or without C/EBPα-p42

		IC_{50} (nM)	
Group	Compound	PDC1#	PDC2#
Vector	Quizartinib	1957±219	1633±120
	Gilteritinib	235.4±5.2	590.6±16.8
C/EBPα-p42	Quizartinib	7588±851	4857±739
	Gilteritinib	1032±14	1526±79

Supplementary Table 3.
IC_{50} of quizartinib and gilteritinib in PDC with or without guanfacine

		IC_{50} (nM)	
Group	Compound	PDC3#	PDC4#
DMSO	Quizartinib	33426±3766	24241±1413
	Gilteritinib	1076±201	768±32
Guanfacine	Quizartinib	7572±409	5166±464
	Gilteritinib	347±78	117±14
